# Room‐Temperature Operable, Fully Recoverable Ethylene Gas Sensor via Pulsed Electric Field Modulation

**DOI:** 10.1002/advs.202500389

**Published:** 2025-03-24

**Authors:** Zeyu Zhang, Bolang Cheng, Yong Zhang

**Affiliations:** ^1^ School of Physics and Optoelectronics Xiangtan University Xiangtan 411105 P. R. China; ^2^ Hunan Institute of Advanced Sensing and Information Technology Xiangtan University Xiangtan 411105 P. R. China

**Keywords:** ambient gas detection, carbon‐based thin‐film transistor, ethylene gas sensor, fully recoverable characteristics, pulsed gate‐voltage modulation

## Abstract

Ethylene (C_2_H_4_) is an important plant hormone, and its concentration can be used as an essential indicator of fruit quality. However, C_2_H_4_ is a non‐polar gas with a relatively stable structure, making it challenging to detect and desorb without heating or irradiation. Here, a pulsed electric field modulation mode for non‐polar gas detection is proposed, which enables fast and complete recovery of sensors at room temperature. Compared to the nearly impossible desorption without electric field assistance, the recovery time for 9 ppm C_2_H_4_ can be reduced to 78 s when the +60 V pulse gate voltage is applied, which is nearly equivalent to the recorded values under heating or irradiation (50 s under 250 °C). Most crucially, with the help of a gate‐induced electric field, the sensor achieves complete desorption within 100 s. This work offers a new approach for fast non‐polar gas detection at room temperature and on‐chip integration of gas sensors.

## Graphical abstract

Utilizing the electric field to regulate the electron behavior of sensing materials, a fast and complete recovery carbon‐based thin‐film transistor ethylene gas sensor is proposed.

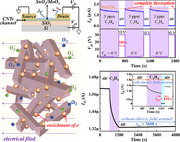

## 1 Introduction

Ethylene (C_2_H_4_) is an achromatic gas with a sweet odor that plays a crucial indicator in fruit ripening.^[^ ^1^, ^2^ ^]^ It is essential to real‐time monitor the concentration of C_2_H_4_ within fruit transportation and storage to ensure fruit quality.^[^ ^3^, ^4^ ^]^ Currently, the main challenges in real‐time monitoring of ethylene are the detection limit of ppm‐level concentration (usually 1–100 ppm during the process of fruit ripening) and the response/recovery time of sensors, mainly due to the difficulty of adsorption and desorption of non‐polar molecules caused by the stable structure of C_2_H_4_ molecules. C_2_H_4_ detection based on the spectroscopic method has the fastest response and recovery time because there is no adsorption and desorption process, but this method requires high cost, large volume, and is inconvenient to use.^[^ ^5^, ^6^, ^7^ ^]^ In contrast, chemoresistive C_2_H_4_ sensors are attracting attention and are expected to be used for real‐time monitoring since they are small, easy to operate, and low‐cost.^[^ ^8^ ^]^ However, the adsorption of C_2_H_4_ is chemisorption, which makes it almost impossible for spontaneous desorption and usually needs to be heated (∼150–450 °C)^[^ ^9^, ^10^, ^11^ ^]^ or irradiated.^[^ ^12^, ^13^ ^]^ Even after integrating the heating or irradiation systems, the average recovery time of reported C_2_H_4_ gas sensors is still >100 s,^[^ ^14^, ^15^, ^16^, ^17^, ^18^ ^]^ and it is still difficult to achieve complete recovery. The incomplete recovery of the gas sensor will lead to the drift of the baseline and the gradual reduction of the response,^[^ ^19^, ^20^ ^]^ which will affect the accuracy of subsequent tests. On the other hand, the integration of heating or irradiation devices also causes difficulties in chip integration and applications. The development of a C_2_H_4_ gas sensor that can recover quickly or even completely without heating and irradiation is particularly important for integrated, portable, and real‐time monitoring applications.

Over the past few years, since the electronic behavior regulation ability of electric fields and the simplicity of on‐chip integration, utilizing electric fields instead of heating or irradiation in gas sensors has attracted widespread attention.^[^ ^21^, ^22^ ^]^ For instance, Wang et al. enhanced the adsorption of polar NH_3_ molecules through the electrostatic interaction of an electric field, achieving a response of 100% to 100 ppb NH_3_ at room temperature.^[^ ^23^ ^]^ Further structural optimizations can adjust the electric field direction and further enhance the selectivity, such as the double‐gate FET sensor with the control gate and the floating gate, the sensor’s response to NO_2_ is more than 35 times that of other interfering gases (NH_3_, SO_2_, CO_2_, etc.).^[^ ^24^ ^]^ The previous studies have utilized electric fields to achieve lower detection limits, higher response, and better selectivity in gas sensors, they also give a potential in principle that the electric field can be utilized to improve the sensors’ recovery speed through rational design. The transmission speed of electric fields in sensing materials approaches the speed of light, allowing for almost instantaneous regulation electronic behavior that enables quicker recovery of gas sensors, particularly when compared to thermal transmission. However, previous works have focused on improving sensor performance by using electric fields to directly influence polar gas molecules such as NO_2_ and NH_3_, but for non‐polar gas molecules, the electric field cannot directly influence the molecules to facilitate desorption. So, it is crucial to clarify why the desorption of C_2_H_4_ is challenging and to devise an appropriate electric field regulation strategy to develop a fast and fully recoverable C_2_H_4_ gas sensor.

In this work, a carbon‐based field‐effect transistor (FET), which represents the cutting edge of current international research, is utilized to fabricate a C_2_H_4_ gas sensor with an exposed channel, and a gate‐induced electric field regulation methodology is developed to replace heating and irradiation. The C_2_H_4_ gas sensor can accumulate electrons on the surface of the gas‐sensing materials through electrostatic induction and significantly increases the probability of electrons being captured by O_2_ molecules, which, in turn, enhances the sensor's recovery rate at room temperature. Through applying pulsed gate voltage, the recovery time can be reduced from almost impossible desorption to tens of seconds and equivalent to the reported record value of those heating or irradiation assisted (∼50 s under 250 °C).^[^ ^9^ ^]^ Above all, the sensor can achieve complete recovery within 100 s while still maintaining excellent linearity, consistency, stability, and selectivity. The proposed method presents a promising approach to utilizing the inherent architecture of the chip to enhance gas sensors’ performance, eliminating the need for on‐chip integration of heating elements or illuminants, and this advancement will provide a crucial step toward achieving on‐chip integration and miniaturization of gas sensors.

## 2 Preparation and Characterization of SnO_2_/MoO_3_ Carbon‐Based FET C_2_H_4_ Sensor

**Figure 1:**
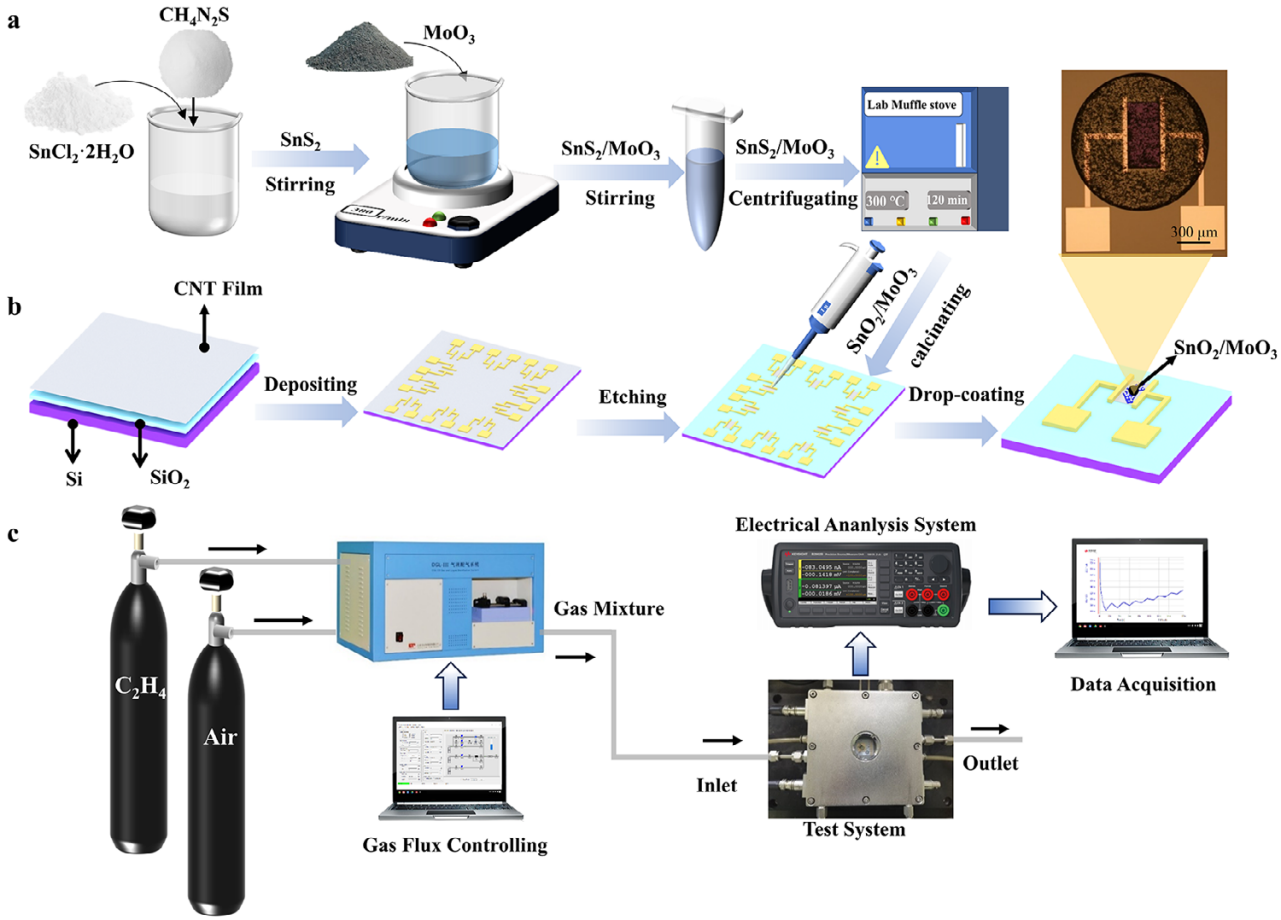
Synthesis process of sensing materials and the gas sensing measurement platform. a) Schematic diagram of the synthesis process of SnO_2_/MoO_3_ composites. b) Schematic diagram of the fabrication process of SnO_2_/MoO_3_ FET sensor and its optical photograph (inset at right top). c) Schematic diagram of the gas sensing measurement platform.

To investigate the challenges of desorbing non‐polar gas molecules and the influence of electric fields on their detection, C_2_H_4_ is selected as the focus of our research due to its stable molecular structure. Owing to its high surface oxygen vacancy density and superior electron sensitization effect, the SnO_2_/MoO_3_ composite is preferred as a gas‐sensing material for C_2_H_4_ adsorption. As shown in **Figure** **1**a, the synthesis of the SnO_2_/MoO_3_ composite involves two main steps: 1) Synthesizing SnS_2_ using a wet chemical method by mixing SnCl_2_·2H_2_O and CH_4_N_2_S. 2) Creating the SnO_2_/MoO_3_ composite through calcination, where SnS_2_ is mixed with MoO_3_ by stirring and then subjected to calcination in a muffle furnace (detailed synthesis processes are shown in *the Experimental Section*). In view of the excellent conductivity and electron transport properties of carbon nanotubes (CNTs), as well as the adjustable band structure in an electric field, an FET‐type sensor is designed based on advanced carbon‐based wafers. As shown in Figure 1b, the SnO_2_/MoO_3_ FET sensor is successfully prepared by three steps: electrode deposition, etching, and drop coating of SnO_2_/MoO_3_ (detailed preparation processes are shown in the *Experimental Section*). To rigorously evaluate the gas‐sensing performance of the sensor, a comprehensive gas‐sensing test platform is established (Figure 1c). This platform consists of two main components: the gas distribution system and the intelligent analyzer. The gas distribution system ensures precise delivery of the required gas concentrations via computerized control, ensuring experimental accuracy. The intelligent analyzer monitors the current variations of the sensor and applies a gate‐induced electric field through computerized control to investigate the impact of electric fields on the gas‐sensing performance.

**Figure 2:**
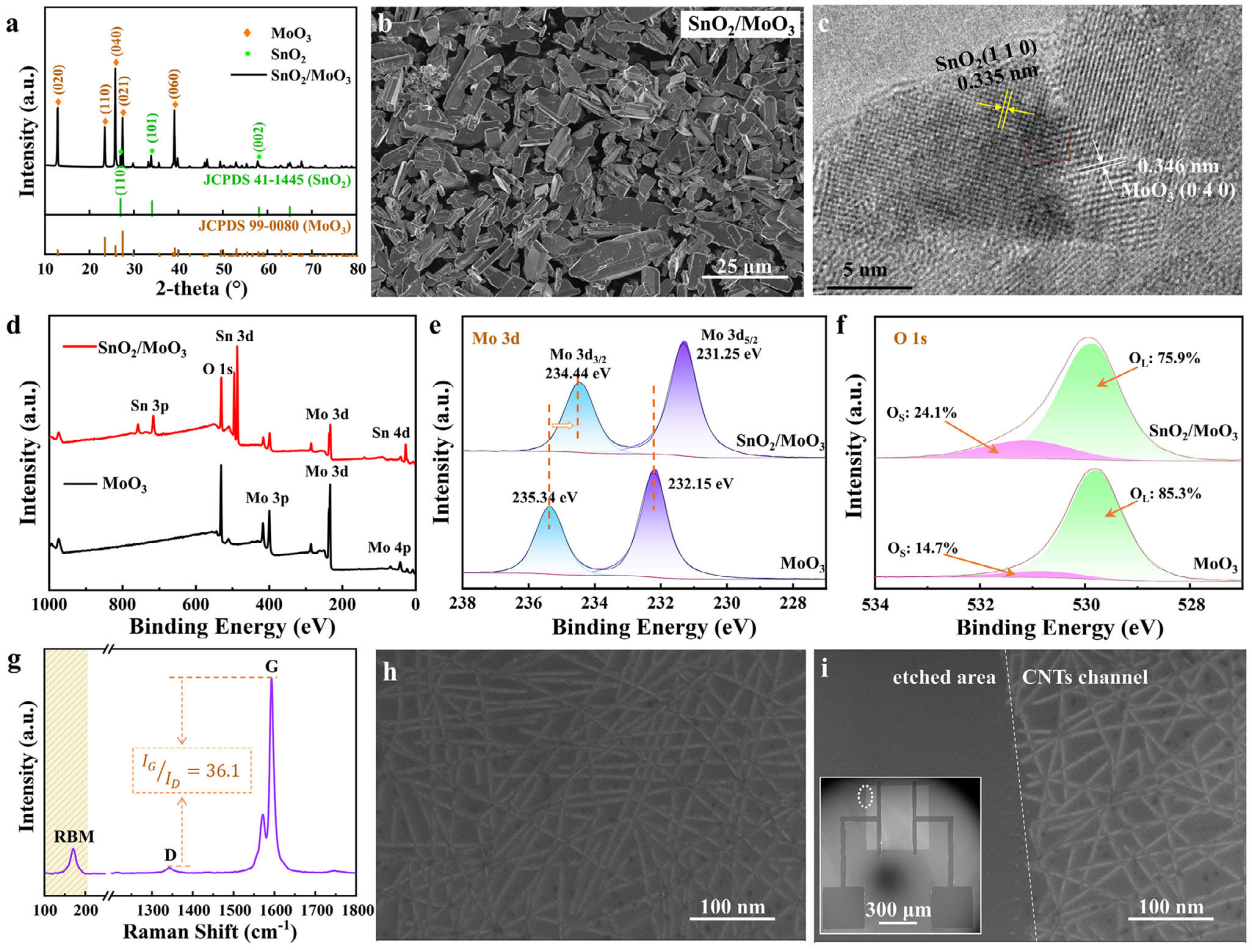
The characterization of SnO_2_/MoO_3_ composites and CNTs channel. a) XRD patterns of SnO_2_/MoO_3_ composites. b) FE‐SEM image of SnO_2_/MoO_3_. c) HRTEM image of SnO_2_/MoO_3_. d) The survey XPS spectra of SnO_2_/MoO_3_ and MoO_3_. High‐resolution XPS spectra e) of Mo 3d and f) O 1s. g) Raman spectrum of the CNTs film. FE‐SEM images of h) CNT film and i) CNTs after etching, the left bottom corner is the FE‐SEM image of a carbon‐based FET.

As shown in **Figure** **2**a, the XRD peaks indicate that the SnO_2_/MoO_3_ composite contains two parts: the orthorhombic MoO_3_^[^ ^25^ ^]^ and the tetragonal rutile structure of SnO_2_, corresponding to standard card JCPDS (99‐0080) and standard card JCPDS (41‐1445), respectively. Additionally, the field emission scanning electron microscopy (FE‐SEM), the energy dispersive spectrometer (EDS) elemental mapping, and the high‐resolution transmission electron microscopy (HRTEM) image of SnO_2_/MoO_3_ in Figure 2b,c and Figure S1 (Supporting Information) further illustrate the composites form and the elemental composition, where SnO_2_ nanoparticles are attached to the surface of MoO_3_ microsheets. The lattice fringes of 0.335 and 0.346 nm correspond to the (1 1 0) plane of tetragonal SnO_2_ and the (0 4 0) plane of orthorhombic MoO_3_, respectively. The lattice distortion at the interface further confirms the presence of SnO_2_ and MoO_3_ and their assembly. Simultaneously, in Figure 2d, the XPS survey spectra are used to analyze the chemical binding states of each element of the SnO_2_/MoO_3_ sensing material. The Sn 3d_3/2_ (494.9 eV) and the Sn 3d_5/2_ (486.5 eV, Figure S2, Supporting Information) in SnO_2_/MoO_3_ belong to Sn^4+^, which is also consistent with previous reports.^[^ ^26^, ^27^ ^]^ Figure 2e shows the locally enlarged spectra of Mo 3d, it contains Mo 3d_3/2_ (234.44 eV) and Mo 3d_5/2_ (231.25 eV). It can be concluded that Mo element is Mo^6+^,^[^ ^28^ ^]^ and the position of Mo 3d peak will move to the direction of lower binding energy after modified SnO_2_. This minor change in the electronic structure of Mo can be attributed to the electron transfer between SnO_2_ and MoO_3_,^[^ ^29^ ^]^ which plays a vital role in the gas‐sensing properties of nanocomposites. The O 1s high‐resolution spectral of MoO_3_ (Figure 2f) can extract two peaks corresponding to lattice oxygen (O_L_ in the figure) and surface adsorbed oxygen (O_S_ in the figure). The content of O_S_ has a significant impact on the gas‐sensing properties of the material.^[^ ^30^ ^]^ After loading SnO_2_, the content of O_S_ increases from 14.7% to 24.1%, which will greatly improve the gas sensing performance of MoO_3_. This improvement is further supported by electron paramagnetic resonance (EPR) analysis, which demonstrates a high concentration of surface oxygen vacancies (O_V_) in the SnO_2_/MoO_3_ composite (Figure S3, Supporting Information). These vacancies not only corroborate the successful synthesis of the composite but also provide active sites for gas adsorption.

For a FET gas sensor, the quality of channel materials is an important factor that influences its sensitivity and response.^[^ ^31^ ^]^ Figure 2g illustrates the Raman spectroscopy of CNTs, and the peaks at 1340 and 1590 cm^−1^ represent the D peak and G peak of CNTs, respectively.^[^ ^32^ ^]^ The high *I*_G_/*I*_D_ ratio of 36.1 indicates a lower defect concentration in CNTs,^[^ ^33^, ^34^ ^]^ which ensures that carbon‐based FET devices have good electrical properties, such as a high on‐off current ratio. The distinct radial breathing mode (RBM) peak in Figure 2g confirms the single‐walled characteristic of the CNTs,^[^ ^35^ ^]^ and these CNTs demonstrate p‐type semiconducting properties (Figure S4, Supporting Information). By combining the RBM (at 170 cm^−1^) and the formula *ω* = 248/*d* (where *ω* and *d* represent RBM Raman shift and CNT diameter, respectively),^[^ ^36^, ^37^, ^38^ ^]^ the average diameter of CNTs can be calculated as about 1.5 nm. On a microscopic level, the CNTs are randomly, uniformly, and densely distributed on the Si/SiO_2_ substrate (Figure 2h). In the preparation of macroscopic devices, CNTs on the Si substrate can be considered as a thin CNT film with an average thickness ≈3 nm) and uniform distribution. This film structure eliminates the need for complex techniques, such as focused ion beam (FIB) processing, to locate and manipulate individual CNT, making the processing easier and more flexible. By employing universal photolithography and reactive ion etching, redundant CNTs located outside the channel are etched to prevent leakage and electrical cross‐talk between devices. As illustrated in Figure 2i, there is a distinct boundary between the channel (right side) and the etched region (left side). On the left side, there are no CNTs, while the CNTs on the right side comprise the channels.

## 3 Research on Performance of SnO_2_/MoO_3_ Carbon‐Based FET C_2_H_4_ Sensor

### 3.1 Mechanism of Electric Field‐Enhanced Desorption of Non‐Polar Gas Molecules

**Figure 3:**
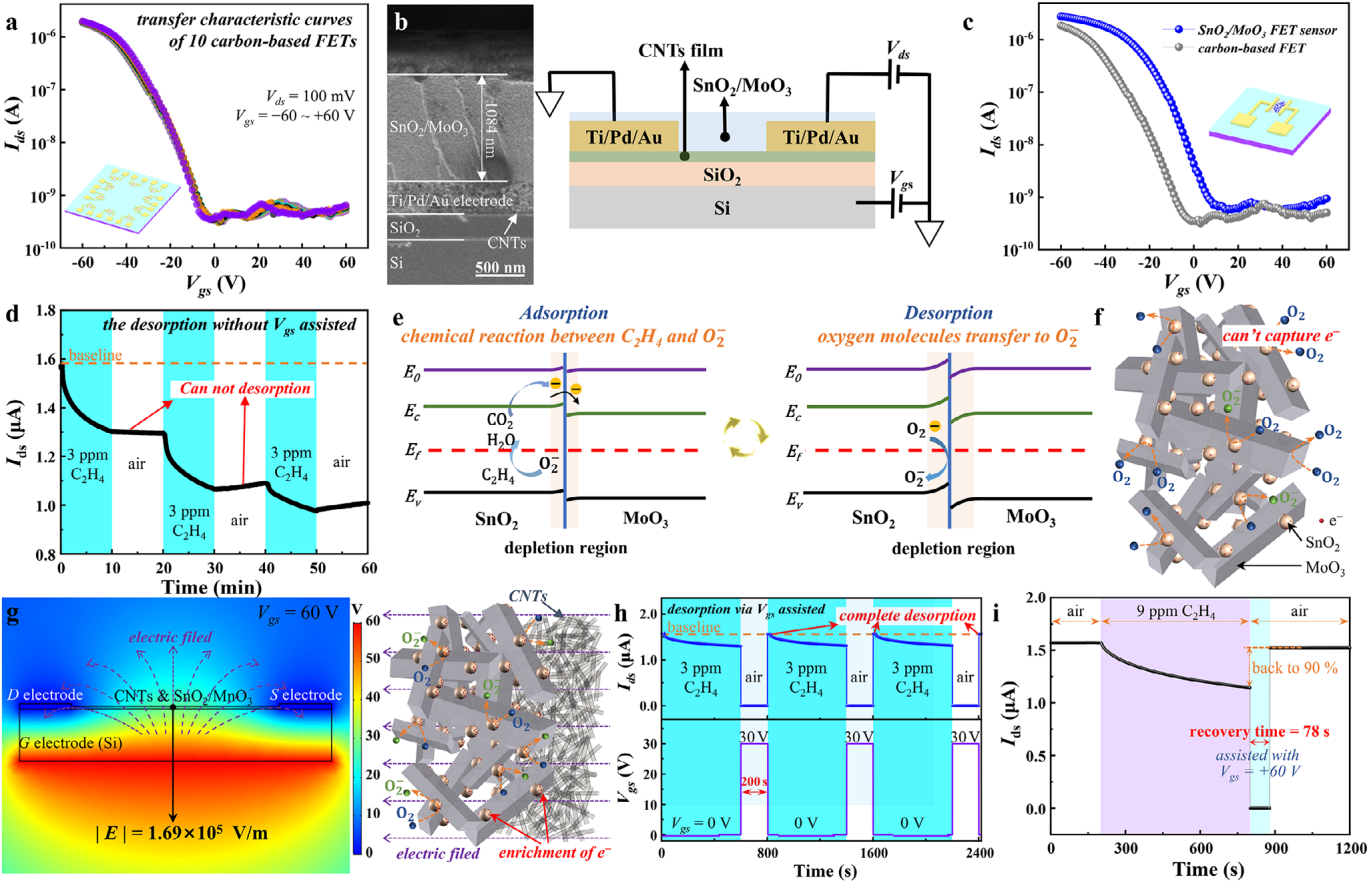
The characterization of SnO_2_/MoO_3_ FET sensor and its sensing mechanism. a) Transfer characteristic curves of 10 carbon‐based FETs. b) FE‐SEM image of cross‐section and the structure schematic diagram of the SnO_2_/MoO_3_ FET sensor. c) Transfer characteristic curve of the SnO_2_/MoO_3_ FET sensor. d) Dynamic response and recovery of the SnO_2_/MoO_3_ FET sensor to 3 ppm C_2_H_4_ at room temperature and without pulsed *V_gs_* assistance. e) Schematic illustration of C_2_H_4_ sensing mechanism of SnO_2_/MoO_3_ composite. f) Schematic illustration of the low probability of O_2_ capturing electrons from SnO_2_/MoO_3_. g) Potential distribution and the schematic illustration of high surface electron density of SnO_2_/MoO_3_ under an electric field, increasing the probability of O_2_ capturing electrons. h) Dynamic response and recovery of the SnO_2_/MoO_3_ FET sensor to 3 ppm C_2_H_4_, where *V_gs_* equals +30 V in the desorption process. i) Response and recovery process to 9 ppm C_2_H_4_.

As the basis of the SnO_2_/MoO_3_ FET sensor, the carbon‐based FET should have excellent repeatability, stability, etc. As shown in **Figure** **3**a, the consistency of carbon‐based FETs is studied by testing and comparing the transfer characteristic curves of ten carbon‐based FETs fabricated on a 1 cm × 1 cm Si wafer from the same batch. Under the condition of *V*_gs_ = −60 ∼+60 V and *V*_ds_ = 0.1 V, there is no significant difference between the transfer characteristic curves. Meanwhile, the on/off current ratio of carbon‐based FETs can reach 10^4^, which signifies a more pronounced current fluctuation in the sensor upon interaction with the target gas and provides a foundation for preparing high‐sensitivity gas sensors. Then, the SnO_2_/MoO_3_ FET sensor is prepared by depositing the SnO_2_/MoO_3_ composite onto the CNTs channel, and the structure schematic diagram and the SEM image of a cross‐section of the sensor are shown in Figure 3b. From top to bottom, this sensor consists of the SnO_2_/MoO_3_ sensing materials (1080 nm thick), Ti/Pd/Au electrode (316 nm thick), thin CNTs film (∼3 nm thick), the SiO_2_ dielectric layer (300 nm thick), and the Si (20 µm thick) gate electrode. Subsequently, the transfer characteristic curve of the SnO_2_/MoO_3_ FET sensor (Figure 3c) is compared to that of the carbon‐based FET. Influenced by the SnO_2_/MoO_3_, the threshold voltage *V_th_* increases from −28.3 V to −7.4 V (Figure S5, Supporting Information). This is because the work function of the material in the channel is changed after coating SnO_2_/MoO_3_, resulting in a modification of the working function and a shift in *V_th_*.

Figure 3d illustrates the dynamic response of the SnO_2_/MoO_3_ FET sensor to 3 ppm C_2_H_4_ at room temperature with *V_gs_* equals 0 V. Without heating, irradiation, and electric field assistance, the current cannot return to the baseline (the recovery rate is only 2% after 20 min). Since the stable molecular structure, C_2_H_4_ is almost impossible for spontaneous desorption, resulting in reduced accuracy in subsequent tests. To further analyze this phenomenon, the energy band diagram for C_2_H_4_ adsorption and desorption is shown in Figure 3e. After the combination, the composite has been identified as an n‐n heterojunction (Figure S6, Supporting Information). Because the work function of SnO_2_ is lower than that of MoO_3_ (5.05 and 5.94 eV, respectively, Figure S7, Supporting Information), electrons are transferred from SnO_2_ to MoO_3_ until the Fermi level reaches equilibrium, forming a complete electron depletion region.^[^ ^39^ ^]^ Before C_2_H_4_ adsorption, O_2_ molecules are adsorbed on the surface of the SnO_2_/MoO_3_ and capture electrons further transform to $O_{2}^{-}$ (Equation (1), determined by the ambient temperature ∼25 °C).^[^ ^40^ ^]^ The electron density at SnO_2_/MoO_3_ surface is reduced thus forming a wider electron depletion region (the right part of Figure 3e), resulting in an increase in SnO_2_/MoO_3_ resistance. Simultaneously, the holes in the CNTs channel are enhanced due to electrons in CNTs moving toward SnO_2_/MoO_3_ under a larger electron concentration gradient, increasing the *I*_ds_ current. During the adsorption of C_2_H_4_, the C_2_H_4_ moleculars will react with the adsorbed $O_{2}^{-}$ and release CO_2_, H_2_O, and electrons (Equation (2)), and the width of the depletion region is decreased (the left part of Figure 3e). Subsequently, electrons flow into the CNTs channel, and a part of the holes is depleted, resulting in a decreased current. Essentially, the desorption of C_2_H_4_ is when O_2_ molecules capture electrons and transfer them to $O_{2}^{-}$, which is the same as the process before C_2_H_4_ adsorption. In desorption, the surface electron density of SnO_2_/MoO_3_ is very low, and it is difficult for O_2_ to obtain electrons and form $O_{2}^{-}$, resulting in an extremely slow recovery (Figure 3f).

(1) $$O_{2}+2e^{-}\to O_{2}^{-}$$

(2) $$C_{2}H_{4}+3O_{2}^{-}\to 2{\mathrm{CO}}_{2}+2H_{2}O+3e^{-}$$

Using irradiation to increase surface electron density and thus improve the recovery speed of sensors is an effective and feasible method^[^ ^13^, ^41^, ^42^ ^]^; besides, using an electric field can also change the surface electron density of sensing materials based on electrostatic induction. The left part of Figure 3g illustrates the potential distribution of the sensor when a +60 V *V*_g_ *_s_* is applied. From bottom to top, the potential of the sensor gradually decreases, reaching only 30 V at the CNTs channel, corresponding to an electric field strength of 1.69 × 10^5^ V m^−1^. The potential distribution and electric field strengths under other *V*_gs_ are shown in Figures S8 and S9 (Supporting Information). As shown in the right part of Figure 3g, when a high electric field is applied to the SnO_2_/MoO_3_ composite, the free electrons of the SnO_2_/MoO_3_ are gathered on the surface of the composite due to electrostatic induction, and the electric field's modulation of the bandgap. A higher surface electron density makes it easier for O_2_ molecules in the air to capture electrons on the surface of SnO_2_, and the probability of O_2_ molecules turning into $O_{2}^{-}$ significantly increases, resulting in a faster recovery speed of the sensor. Therefore, applying a high electric field in the desorption process can significantly enhance the recovery capability of the SnO_2_/MoO_3_ FET sensors at room temperature.

Figure 3h illustrates the dynamic response of the SnO_2_/MoO_3_ FET sensor to 3 ppm C_2_H_4_ at room temperature and with *V*_gs_ equals +30 V in the desorption process. When 3 ppm of C_2_H_4_ is injected into the test chamber, the *I*_ds_ gradually decrease. Upon introducing air while simultaneously applying a *V*_gs_ of +30 V, the FET channel closes, causing *I*_ds_ to drop to zero. It is important to note that the process of introducing air and applying *V*_gs_ occurs synchronously, and it is not instantaneous desorption of C_2_H_4_ when *I_ds_* converts to zero, but gradual desorption occurs over a period of 200 s. In detail, the entire C_2_H_4_ gas detection process can be divided into two steps: (I) The C_2_H_4_ is introduced into the chamber, and the *V*_gs_ is set as 0 V. In this step, the current decreases continuously due to the depletion between holes and electrons that are released from the chemical reaction between C_2_H_4_ and $O_{2}^{-}$ (Equation (2)); (II) The air is introduced into the chamber, and *V*_gs_ is adjusted from 0 to +30 V simultaneously. In this step, the current is almost 0 A because the channel narrows, and a higher surface electron density is obtained by electrostatic induction that can enhance the probability of O_2_ molecules capturing electrons. In contrast to the nearly impossible desorption without electric field assistance, the sensor can achieve complete desorption (the current recovers to the baseline level) in 200 s at room temperature. Actually, the duration of +30 V *V*_gs_ applied will also affect the recovery rate, as shown in Figure S10 (Supporting Information), only 48.9% reached at 50 s. According to the calculation principle, the recovery time can be defined when the recovery rate equals 90%, and the recovery time of 3 ppm C_2_H_4_ is 150 s (Figure S11, Supporting Information). The recovery time of 9 ppm C_2_H_4_ is just 78 s when +60 V pulsed *V*_gs_ is applied (Figure 3i), which is nearly equivalent to the recorded values under heating or irradiation assistance (**Table** **1**, 50 s under 250 °C^[^ ^9^ ^]^). To facilitate understanding of the impact of pulsed *V*_gs_ on full recovery time, Figure S12 (Supporting Information) illustrates the relationship between recovery time and pulsed *V*_gs_. With a +60 V pulsed *V*_gs_ applied, the sensor fully recovers to its baseline in just 100 s.

**Table 1:** Comparison of the recovery time of this work with some previous C_2_H_4_ gas sensors.

Materials	Structure	Concentration	Response	Experiment Condition	Recovery time	Ref.
Pd‐SnO_2_	resistor	20 ppm	100%	250 °C	103 s	[14]
Pd/rGO/α‐Fe_2_O_3_	resistor	10 ppm	900%	250 °C	50 s	[9]
SnO_2_	resistor	8 ppm	121%	350 °C	144 s	[15]
WO_3_–SnO_2_	resistor	6 ppm	∼65%	300 °C	∼500 s	[16]
SnO_2_	resistor	25 ppm	550%	350 °C	678 s	[17]
Pd‐V_2_O_5_‐TiO_2_	resistor	1 ppm	18.9%	325 °C	∼1000 s	[18]
Cr_2_O_3_‐SnO_2_	resistor	2.5 ppm	1210%	375 °C	69 s	[43]
ZnO Flakes	resistor	29 ppm	8.6%	RT	480 s	[44]
ZnO‐Ag	resistor	50 ppm	7%	RT	980 s	[45]
SWCNTs‐Pd NPs	resistor	10 ppm	40%	RT	‐	[46]
SWCNT @PdCl_2_ catalytic mixture	FET	20 ppm	20%	RT	∼240 s	[2]
Pd/P_3_HT	FET	25 ppm	18.5%	RT	∼600 s	[47]
SnO_2_/MoO_3_	FET	3 ppm 9 ppm	16.8% 27.2%	RT	∼150 s (pulsed *V*_gs_ = +30 V) ∼78 s (pulsed *V*_gs_ = +60 V)	Our work

RT denotes room temperature.

### 3.2 Performance of Sensors Assisted by Electric Fields and Their Applications

**Figure 4:**
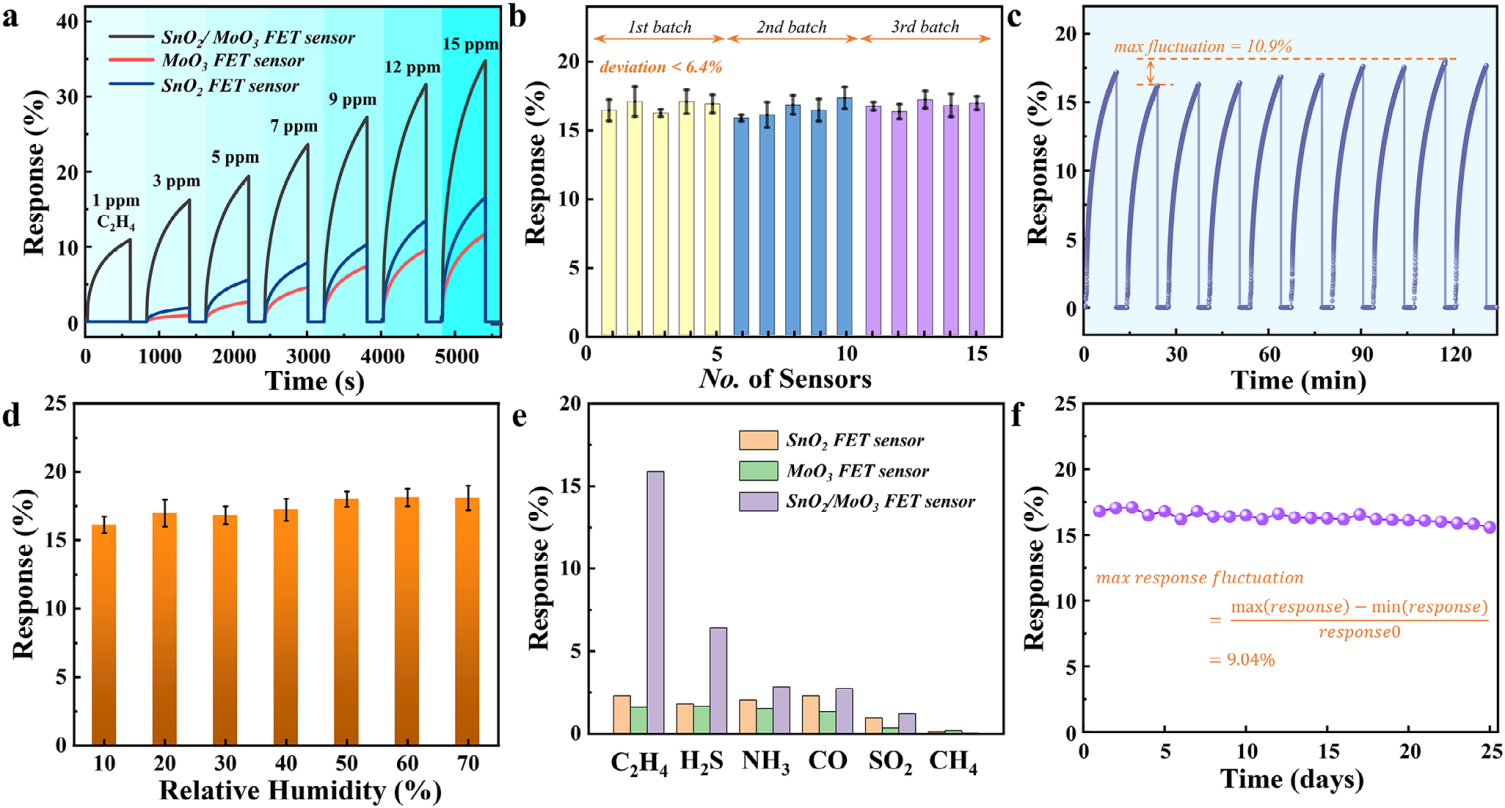
The consistency, selectivity, long‐term stability, and other key characteristics of SnO_2_/MoO_3_ FET sensor. a) Real‐time gas response of SnO_2_, MoO_3_, SnO_2_/MoO_3_ FET sensor toward 1–15 ppm C_2_H_4_ concentration. b) Response values of fifteen sensors that are randomly selected from different fabrication batches. c) Repeating response‐recovery curve of SnO_2_/MoO_3_ FET sensor to 3 ppm C_2_H_4_. d) Influence of humidity in sensor response. Responses of SnO_2_/MoO_3_ FET sensor e) toward different gases of 3 ppm at room temperature and f) toward 3 ppm C_2_H_4_ within 25 days.

The effects of various C_2_H_4_ concentrations (1–15 ppm) on the response properties of MoO_3_, SnO_2_, SnO_2_/MoO_3_ FET sensors, and carbon‐based FET are studied and presented in **Figure** **4**a and Figure S13 (Supporting Information). The relationship between response and C_2_H_4_ concentration is linear, and the sensitivity of the SnO_2_/MoO_3_ FET sensor can reach 1.7%/ppm (Figure S14, Supporting Information). The SnO_2_/MoO_3_ FET sensor demonstrated a significantly higher response and shorter recovery time to C_2_H_4_ compared to others (Figure S15, Supporting Information). This performance improvement is attributed to the well‐defined SnO_2_/MoO_3_ heterostructure, where the interfacial charge transfer facilitates gas adsorption and desorption kinetics (Figure S16, Supporting Information).^[^ ^48^, ^49^ ^]^ Consequently, the SnO_2_ loading capacity plays a critical role in determining sensor performance. As shown in Figure S17 (Supporting Information), the optimal response is achieved at a SnO_2_:MoO_3_ molar ratio of 1:18, indicating that excessive SnO_2_ loading may disrupt the heterostructure. Additionally, as shown in Figure S18 (Supporting Information), the experimental detection limit of this sensor is 800 ppb (response 5.93%), while the theoretical detection limit is 100.25 ppb.

In order to further study the C_2_H_4_ gas sensing mechanism, the transfer characteristic curve of SnO_2_/MoO_3_ FET sensor is tested in air and in 15 ppm C_2_H_4_, respectively (Figure S19, Supporting Information). When the gas in the chamber is converted from air to C_2_H_4_, the transfer characteristic curve shifts to the left, decreasing in Δ*I*_ds_ when the *V*_gs_ is fixed. The above phenomenon can be explained by the relationship between the composite's work function and FET *V_th_*, and the work function is changed by electron transfer caused by gas adsorption on the composite.^[^ ^50^ ^]^ When SnO_2_/MoO_3_ FET sensor is exposed to air, O_2_ will capture electrons from the surface of the gas‐sensing material to form $O_{2}^{-}$, which will also increase the work function. While the sensor is exposed to C_2_H_4_, C_2_H_4_ will decompose into CO_2_ and H_2_O when reacting with the adsorbed $O_{2}^{-}$ on the surface of the material and will release electrons, resulting in a decrease in the work function. According to the positive correlation between *V*_th_ and work function,^[^ ^51^, ^52^ ^]^ the transfer characteristic curve of the SnO_2_/MoO_3_ FET sensor under the action of C_2_H_4_ will be reset. Therefore, even in extremely low concentrations of C_2_H_4_, *I_ds_* can shift from a large current to a very small one, leading to a significant response.

In addition, the reliability and stability of SnO_2_/MoO_3_ FET sensors is investigated. As shown in Figure 4b, the response of 15 SnO_2_/MoO_3_ FET sensors is basically consistent (the response deviation between different batches is <6.4%), where sensors are randomly selected from three batches of devices that are prepared at different times. The repeatability of response is a crucial characteristic for the practical application of a gas sensor. In order to verify the repeatability of response and recovery of the sensor under +30 V pulsed *V*_gs_, 10 repeated dynamic response‐recovery processes to 3 ppm C_2_H_4_ gas are carried out (Figure 4c), and the dynamic response and recovery process of the sensor is stable and reliable (max fluctuation is 10.9%). Meanwhile, Figure 4d illustrates the influence of the humidity on response. Compared with the sensor without pulsed *V*_gs_ assisted (Figure S20, Supporting Information), the sensor with +30 V pulsed *V*_gs_ assisted has good moisture resistance. The response is consistently maintained within the range of 16%–18.2% for relative humidity between 10% RH and 70% RH, and the response deviation remains below 12.1%. This enhancement is attributed to the pulsed electric field‐induced electron accumulation on the SnO_2_/MoO_3_ surface, which neutralizes positively charged water molecules generated via physical/chemical adsorption and dynamically promotes their desorption (Figure S21, Supporting Information). In fruit storage and transport, the CO from automobile exhaust or refrigeration equipment and the CH_4_, SO_2_, NH_3_, and H_2_S from fruit rots may affect the response of the SnO_2_/MoO_3_ FET sensors. Figure 4e illustrates the cross‐sensitivities of these gases in the MoO_3_, SnO_2_, and SnO_2_/MoO_3_ FET sensors. The responses to 3 ppm CH_4_, SO_2_, CO, NH_3_, and H_2_S are minor or zero at room temperature, assuring the high C_2_H_4_ selectivity of the SnO_2_/MoO_3_ FET sensor. The C_2_H_4_ gas sensing selectivity has been greatly improved compared to the other two sensors. Furthermore, long‐term stability is also vital to the gas sensor. As shown in Figure 4f, the absolute response error is 1.52% and the max response fluctuation is just 9.04% within 25 days. Because of the excellent consistency and the preferable long‐term stability, the developed SnO_2_/MoO_3_ FET sensor can be applied to fruit transport and storage in the future.

**Figure 5:**
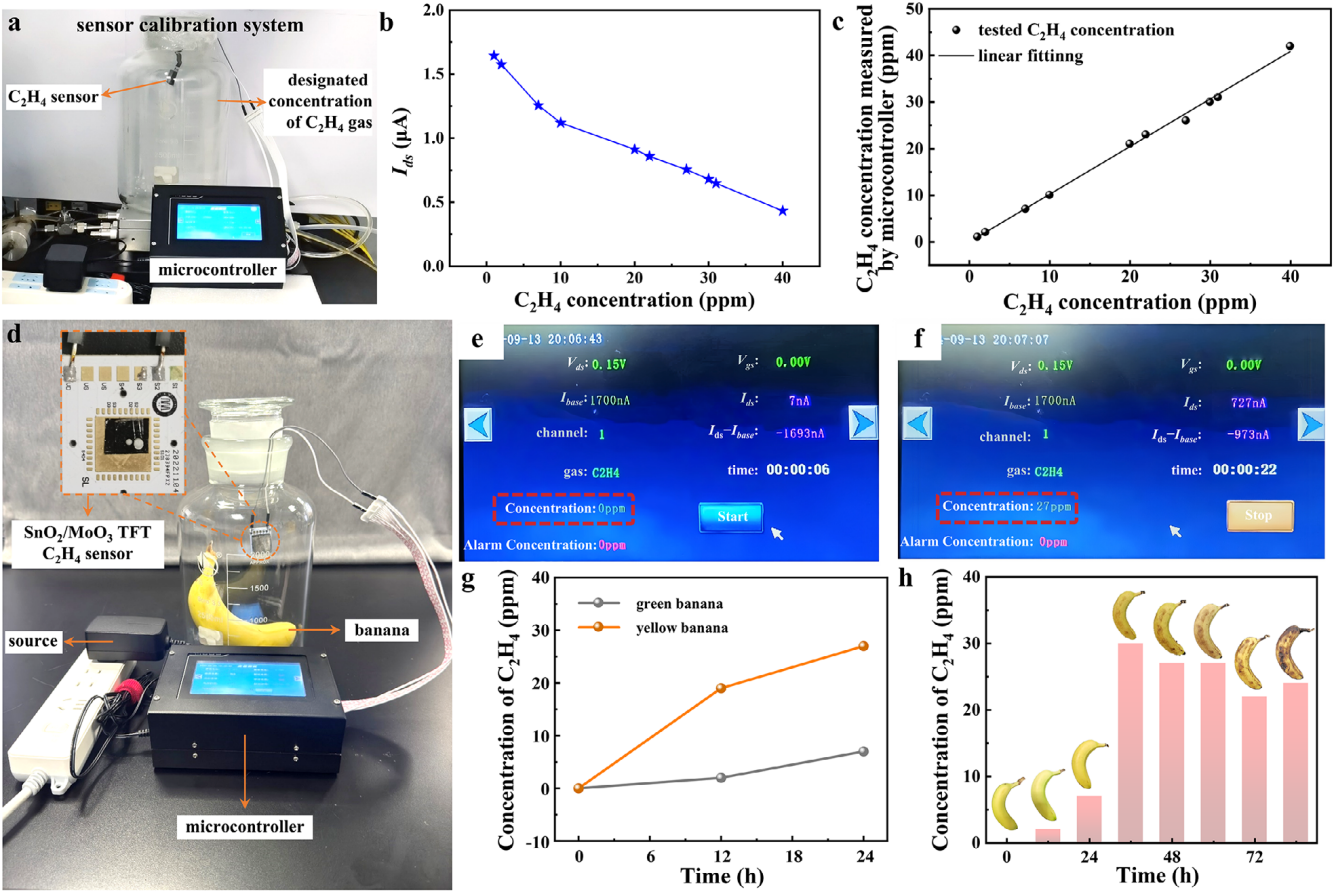
Real‐time C_2_H_4_ concentration monitoring during banana ripening. a) The optical graph of the sensor calibration system. b) The relationship between the *I_ds_* tested by the microcontroller and C_2_H_4_ concentration. c) The relationship between the output C_2_H_4_ concentration and designated C_2_H_4_ gas concentration. d) The optical photograph of the real‐time C_2_H_4_ concentration monitoring system, the inset at the upper left corner illustrates the sensor chip. e, f) The interface displays different storage times (0 and 24 h). g) Comparison of C_2_H_4_ concentration between green and ripe bananas within 24 h. h) Change of C_2_H_4_ concentration during bananas from green to rot.

Lastly, in order to evaluate the sensors’ practical application capability, during banana ripening, a minute‐level real‐time C_2_H_4_ concentration monitoring is conducted by integrating the sensor and microcontroller. As shown in **Figure** **5**a, the C_2_H_4_ concentration monitoring system is calibrated in the specific concentration of C_2_H_4_ gas. Figure 5b delineates the relationship between the *I_ds_* tested by the microcontroller and the specific C_2_H_4_ concentration. As the concentration of C_2_H_4_ escalates, the *I_ds_* exhibit a gradual decline, which is similar to that tested by the electric analysis system. Simultaneously, within the C_2_H_4_ concentration range of 1–40 ppm, the maximum deviation in the C_2_H_4_ concentration output from the microcontroller is less than 5% (Figure 5c). Figure 5d is an overall photo of the actual application. A 120 g banana is placed into a wide‐mouth bottle, which is not sealed to avoid C_2_H_4_ accumulation. The sensor chip is packaged on a printed circuit board (PCB) and placed in the bottle. In this way, the C_2_H_4_ that is released by the banana at different times can be monitored by a portable system. Figure 5e, respectively illustrates the C_2_H_4_ concentration when a banana is just put into the bottle and after 24 h. After 24 h, the concentration of C_2_H_4_ is increased from 0 to 27 ppm. Figure 5g compares the C_2_H_4_ concentration released by bananas in one day, where the concentration of C_2_H_4_ released by ripe bananas is significantly higher than that released by immature bananas. The C_2_H_4_ concentration of different maturation stages and the images of the banana are shown in Figure 5h (the corresponding optical photographs and video are shown in Figure S22 and Movie S1, Supplementary Movie). There is a significant climacteric change in C_2_H_4_ concentration from 24 to 36 h. As a respiratory climacteric fruit, bananas are susceptible to C_2_H_4_ concentration in aging and metamorphosis, and the storage environment needs to be adjusted in time during picking and transportation. The above application provides a new means for real‐time monitoring of C_2_H_4_ concentration in the process of fruit growth, picking, transportation, and storage, which is of great significance for the development of intelligent agriculture.

## 4 Conclusion

In summary, a pulsed electric field‐assisted mode is proposed for FET gas sensors, which can achieve fast and complete desorption of non‐polar gas at room temperature. The prepared SnO_2_/MoO_3_ FET sensor shows a 5.93% response to 800 ppb C_2_H_4_, and a recovery time of just 78 s at a +60 V pulse *V*_gs_ when exposed to 9 ppm C_2_H_4_. The pulsed electric field promotes the recovery of the sensor by regulating the surface electron density of sensing materials so that the prepared SnO_2_/MoO_3_ FET sensor can fully recover the sensor in 100 s without external heating and irradiation. The sensor also has an excellent linear relationship between response and C_2_H_4_ concentration in the range of 1–15 ppm, as well as good repeatability, moisture resistance, and stability. Furthermore, the micro‐nano processing technology can be used to prepare SnO_2_/MoO_3_ FET sensors in batches to ensure the consistency of sensor performance, and the response deviation between different batches of sensors is less than 6.4%. This work will provide a new strategy for detecting non‐polar gas molecules, as well as provide a new method for enhancing the performance of FET gas sensors and enabling on‐chip integration of gas sensors.

## 5 Experimental Section

### Preparation Process of SnO_2_/MoO_3_

SnO_2_/MoO_3_ composite were prepared by wet chemical method and high‐temperature calcination treatment. First, 210 mg SnCl_2_·2H_2_O and 70 mg CH_4_N_2_S were dissolved in 30 mL deionized water and stirred at room temperature for 30 min. Next, 2.4 g MoO_3_ was added to the above solution and stirred at room temperature for 6 h to synthesize SnS_2_/MoO_3_ composite. Finally, SnS_2_/MoO_3_ composite were filtered out from the suspension and washed with deionized water and ethanol twice, and then annealed in a Muffle furnace at 300 °C for 2 h to obtain the final SnO_2_/MoO_3_ sensing material. All chemical reagents used in this work were analytic grade and purchased from Aladdin Biochemical Technology Co., Ltd. (Shanghai, China) without further purification.

### Preparation Process of Carbon‐Based FETs

The preparation of the SnO_2_/MoO_3_ FET sensor was based on a four‐inch CNTs wafer, where CNTs were deposited on the surface of the Si/SiO_2_ wafer by dip‐coating method.^[^ ^53^, ^54^ ^]^ Specifications of the Si wafer include a p‐type doping and a resistivity of 1.5 × 10^3^ Ω cm. The specific process was as follows: First, a 4‐inch wafer was cut into multiple sizes of 1 × 1 cm substrates, and then a laser direct writing lithography machine (MicroWriter ML3) was used to etch the source and drain regions. Subsequently, the Ti/Pd/Au films (Thickness: 6/110/ 200 nm) were deposited on the regions one by one to fabricate S/D electrodes through electron beam evaporation (DE400). In order to avoid mutual interference between different SnO_2_/MoO_3_ FET sensors, redundant CNTs were cut off by photolithography and reactive ion etching (Haasrode‐R200A, China) to form a size of 500 × 600 µm channel. Finally, the prepared SnO_2_/MoO_3_ sensing material was deposited on the CNTs using a micropipette to form SnO_2_/MoO_3_ FET sensors with a channel exposed structure (the SnO_2_/MoO_3_ come into contact with the Ti/Pd/Au electrode).

### Characterization and Gas Sensing Testing Setup

The morphology of CNTs films and SnO_2_/MoO_3_ sensing materials was studied by FE‐SEM (Hitachi SU5000), and the purity of CNTs films was verified by Raman spectroscopy. The X‐ray diffraction (XRD, UItima IV), the HRTEM (Thermo Talos F200i), and the X‐ray photoelectron spectroscopy (XPS, Thermo ESCALAB 250xi) from a Cu Kα radiation source (λ = 0.15418 nm) were used to characterize the crystal structure, elemental composition, and element valence of SnO_2_/MoO_3_ sensing materials. The electrical properties of the carbon‐based FET and SnO_2_/MoO_3_ FET sensors were measured using a Keithly 4200 semiconductor analyzer and a probe platform (Cascade Microtech MPS 150). The performance of the SnO_2_/MoO_3_ FET sensor was investigated by a gas‐sensing measuring platform, which was composed of the DGL‐III gas‐liquid distribution system, CGS‐MT intelligent gas analysis system, and electric analysis system (Keysight B2900). The flux of dry air (79% N_2_ and 21% O_2_) and target gas (Dalian Date Gas Co. Ltd. (Dalian, China)) were controlled by mass flow controllers and then mixed to obtain different concentrations. The relative humidity of the chamber was adjusted via the dual‐flow method (Figure S23, Supporting Information). The response of the sensor was defined as |*I*_g_‐*I*_a_|/*I*_a_×100%, and the recovery rate was calculated by |*I*_g_‐*I*_a_|/|*I*_r_‐*I*_a_|×100%, where *I*_g_ and *I*_a_ were the current of the FET sensor in the target gas and air, respectively, and *I*_r_ was the current of the FET sensor at the specified electrical field applied time.^[^ ^33^, ^55^ ^]^ In all experiments, *V*_ds_ was set to 0.1 V, and the duration for introducing C_2_H_4_ remains constant at 600 s (Figure S24, Supporting Information).

### Statistical Analysis

The analysis of sensor response error bars under varying humidity conditions was based on three samples. Data presentation in the figure was depicted as mean ± standard deviation (SD), where the height of the bar chart represents the mean value and the error bars indicate the SD. Origin was utilized for statistical analysis.

## Conflict of Interest

The authors declare no conflict of interest.

## Author Contributions

Z.Z. and B.C. contributed equally to this work. The manuscript was written through the contributions of all authors. All authors have given approval to the final version of the manuscript.

## Supporting information



Supporting Information

Supplemental Movie 1

## Data Availability

The data that support the findings of this study are available from the corresponding author upon reasonable request.
