# Supplementary material for: Room‐Temperature Operable, Fully Recoverable Ethylene Gas Sensor via Pulsed Electric Field Modulation
Source: Adv Sci (Weinh). 2025 Mar 24;12(19):2500389. doi: 10.1002/advs.202500389 (PMC12097100; doi:10.1002/advs.202500389)
Supplement: Supplementary file 1 — Supporting Information [file ADVS-12-2500389-s002.docx]

Supporting Information

**Room-Temperature Operable, Fully Recoverable Ethylene Gas Sensor via Pulsed Electric Field Modulation**

*Zeyu Zhang^1^†, Bolang Cheng^1^†, Yong Zhang^1,2^****

**This file includes:**

Figure S1 to Figure S24

**Figure S1 to Figure S24**


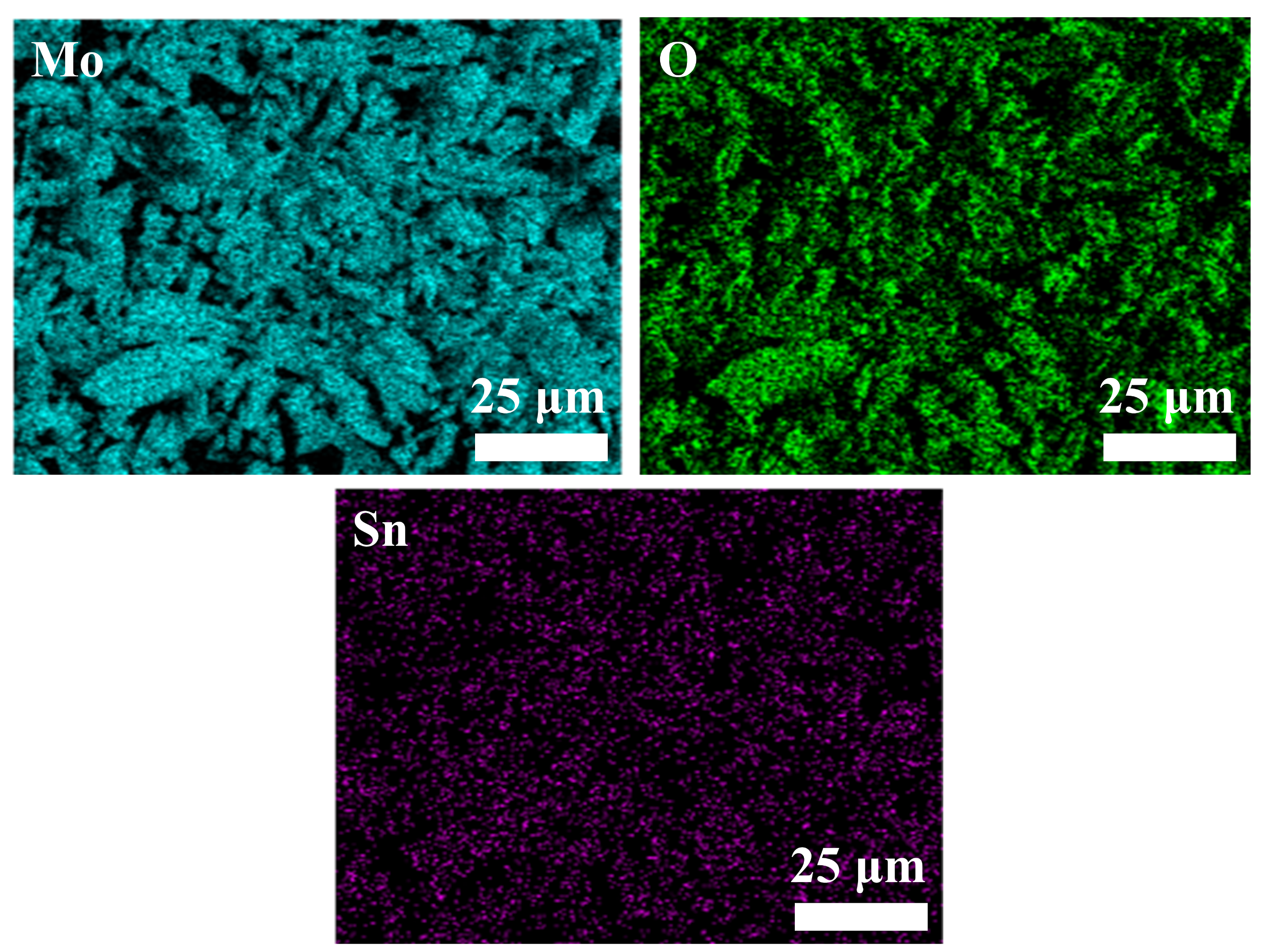


**Figure S1.** **Energy dispersive spectrometer (EDS) elemental mappings of the SnO_2_/MoO_3_ composite material.**


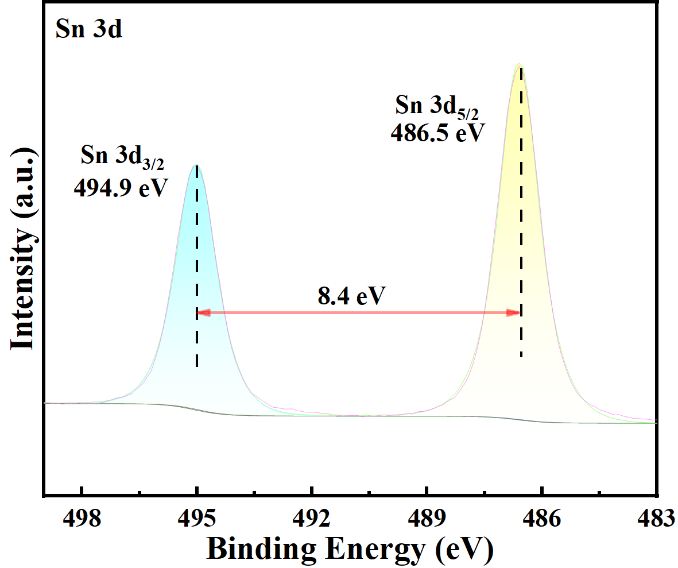


**Figure S2. X-ray photoelectron spectroscopy (XPS) analysis of** **SnO_2_/MoO_3_ composite, focusing on Sn 3d.** The peaks observed are Sn 3d_3/2_ (494.9 eV) and Sn 3d_5/2_ (486.5 eV), with a difference of 8.4 eV between the two peaks, which indicates the presence of Sn^4+^.


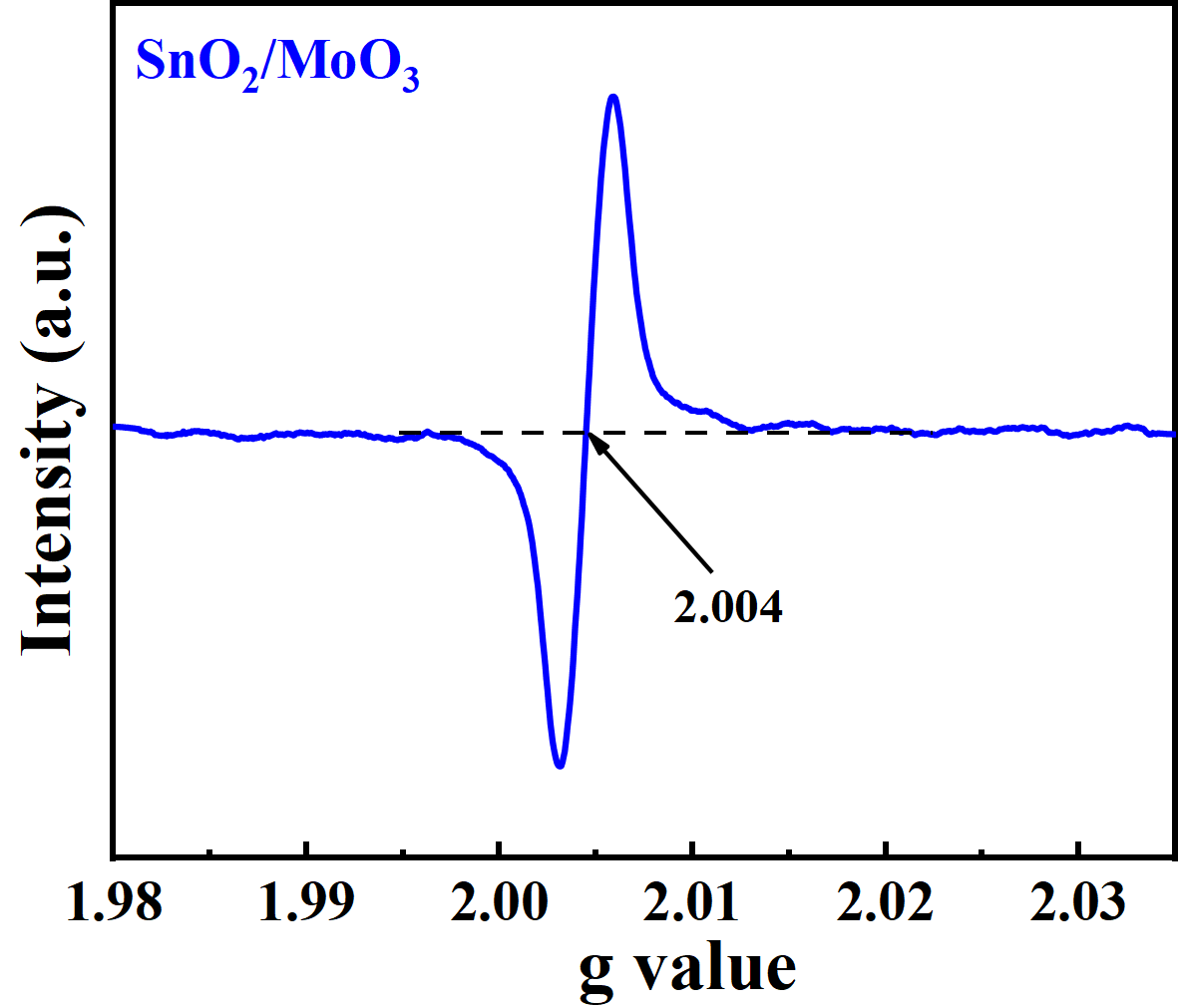


**Figure S3.** **Electron paramagnetic resonance (EPR) spectroscopic characterization of SnO_2_/MoO_3_.** The EPR spectrum of SnO_2_/MoO_3_ exhibits a pronounced signal at g = 2.004, attributable to electron trapping at defect sites. This result confirms a significant concentration of oxygen vacancies on the surface of the SnO_2_/MoO_3_ composite. ^[S1]^


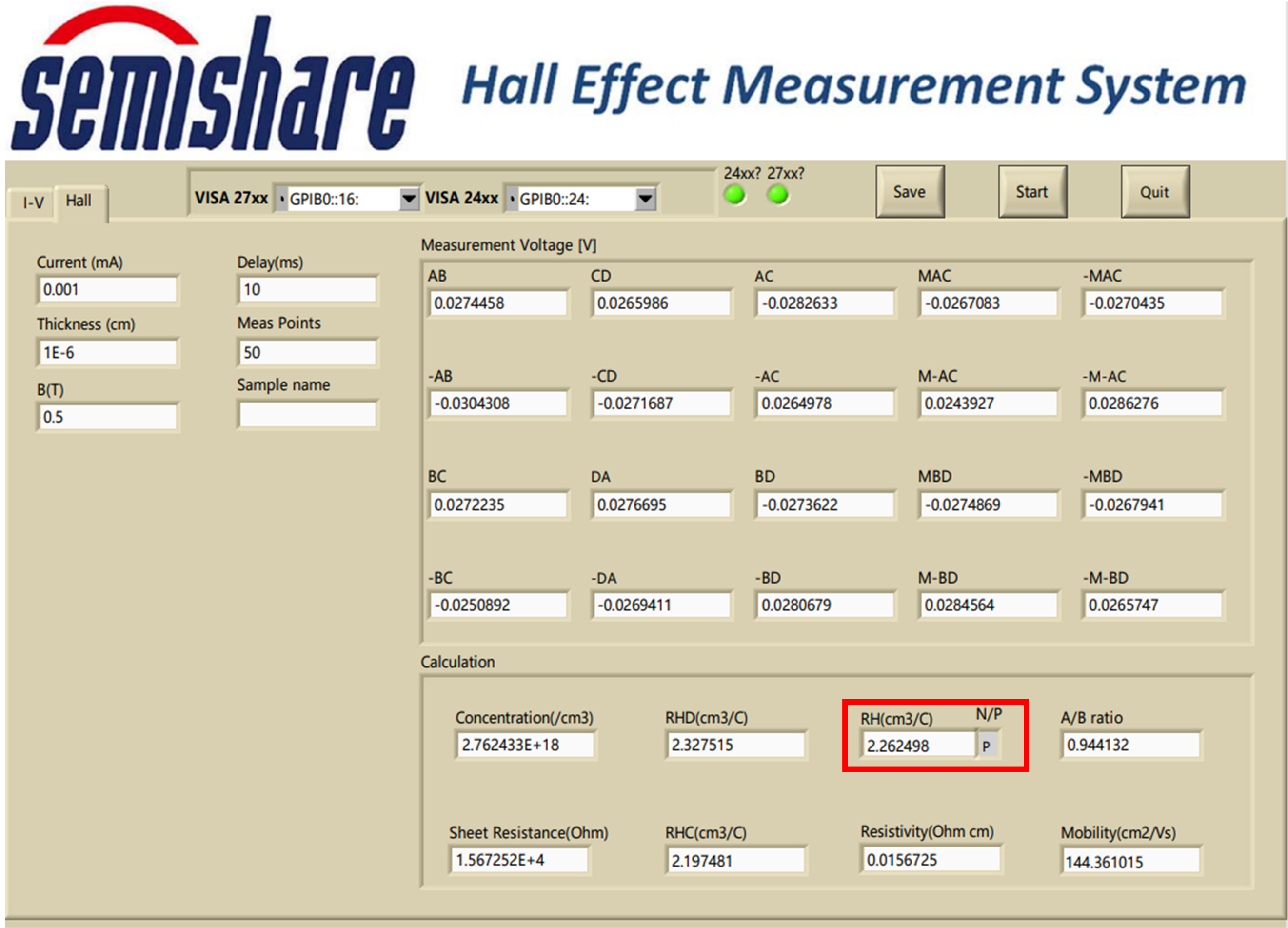


**Figure S4. The Hall effect characterization of CNTs, which reveals that the CNTs exhibit p-type semiconductor behavior.**


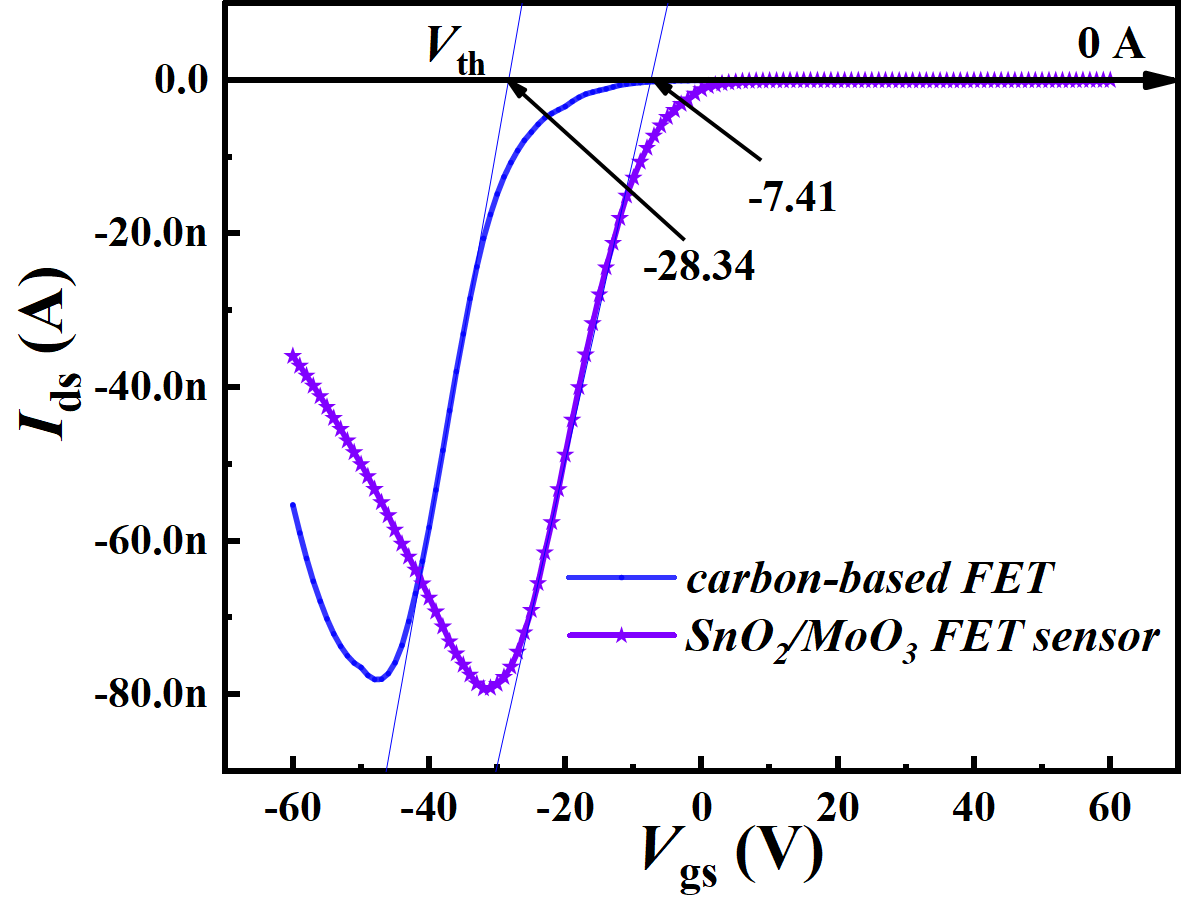


**Figure S5 The threshold voltage of carbon-based FET and SnO_2_/MoO_3_ FET sensor.** The threshold voltage is calculated using the maximum transconductance method. Firstly, the transconductance is calculated across the entire transfer characteristic curve. Then, the point of maximum transconductance is located, and a tangent line is drawn at this point to intersect with the X-axis. The intersection point represents the threshold voltage.


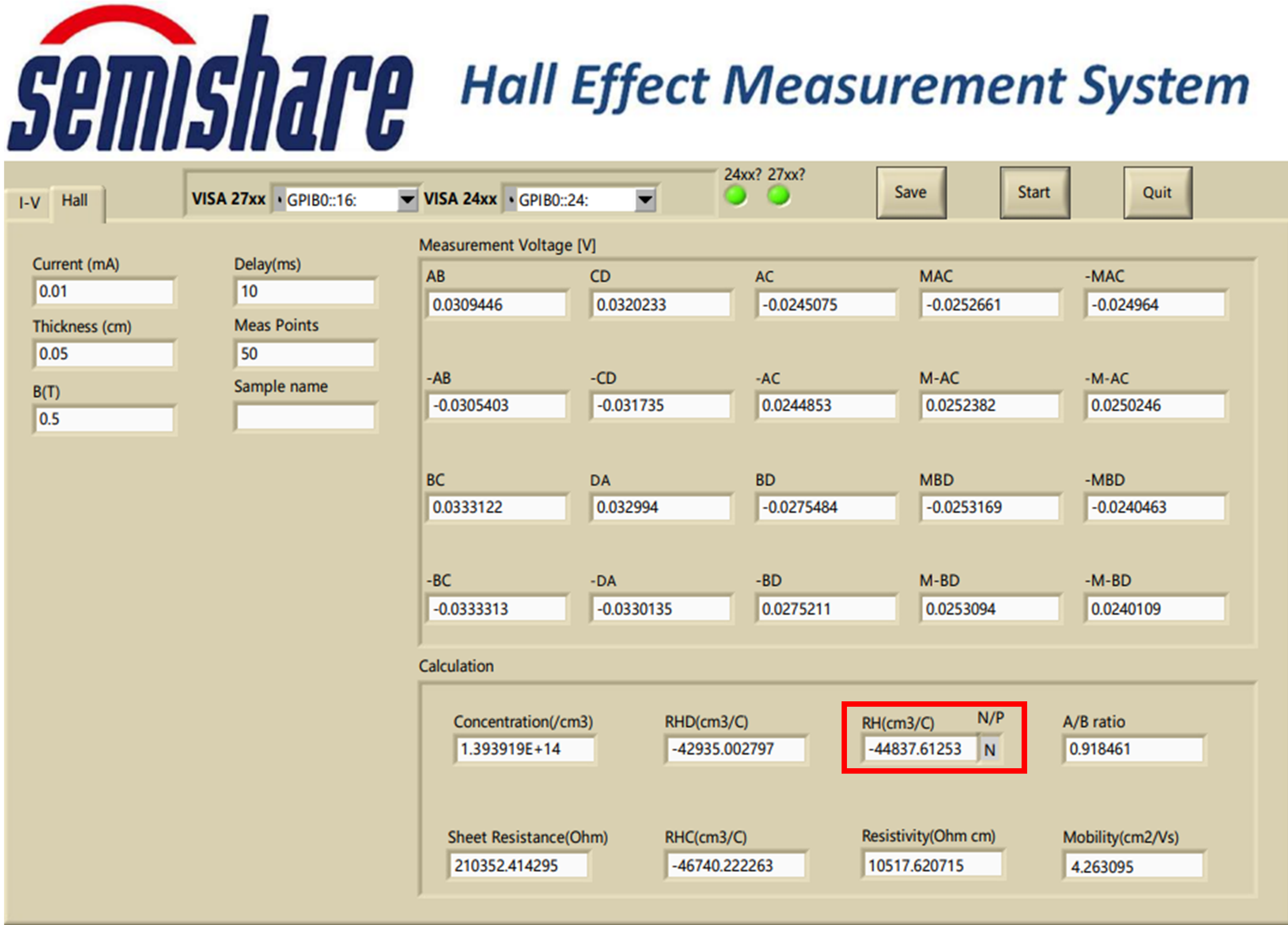


**Figure S6. The Hall effect characterization of SnO_2_/MoO_3_ composite, which reveals that the SnO_2_/MoO_3_ composite exhibits n-type semiconductor behavior.**


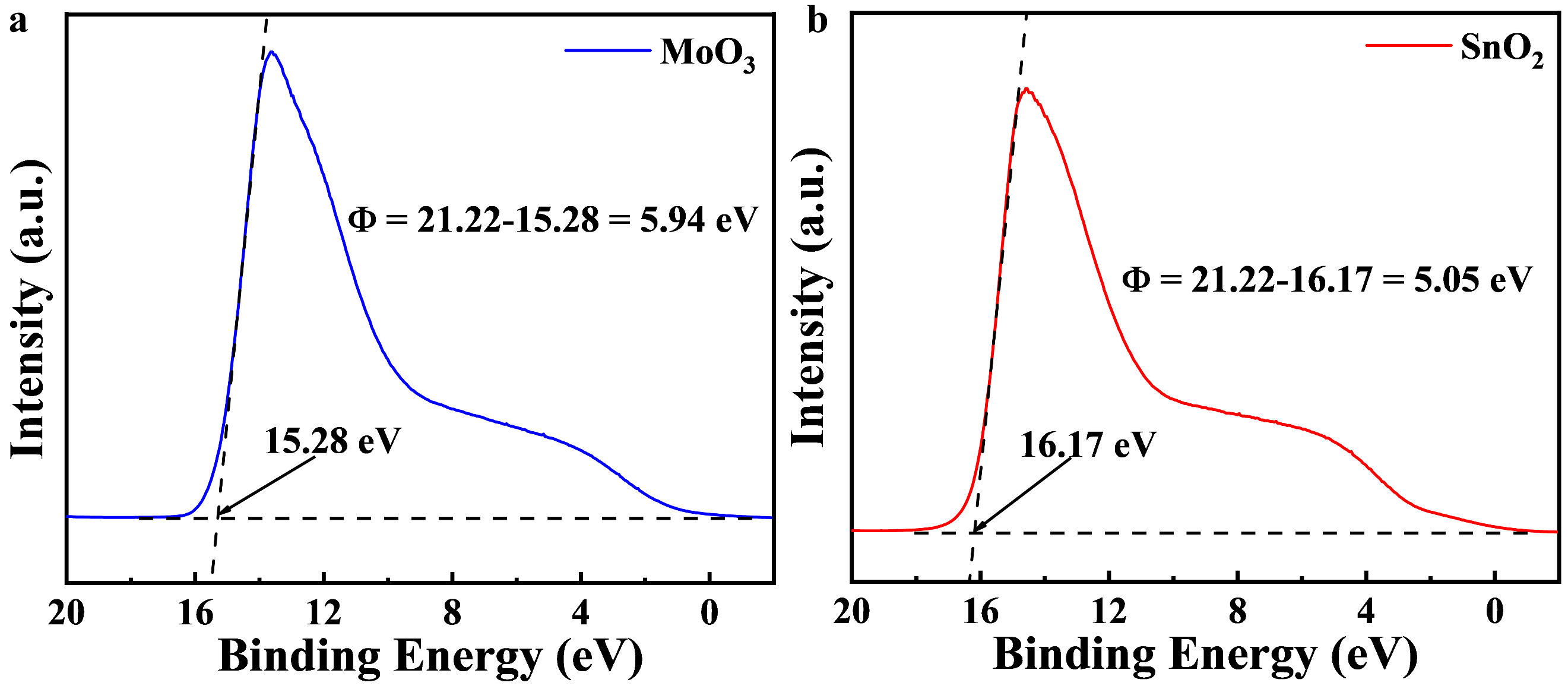


**Figure S7. Ultraviolet photoelectron spectroscopy (UPS) spectra of (a) MoO_3_ and (b) SnO_2_.** The working functions are obtained from the diﬀerence between the incident photon energy (21.22 eV) and the secondary edge. The work functions of pure SnO_2_ and pure MoO_3_ are determined to be 5.05 and 5.94 eV, respectively.


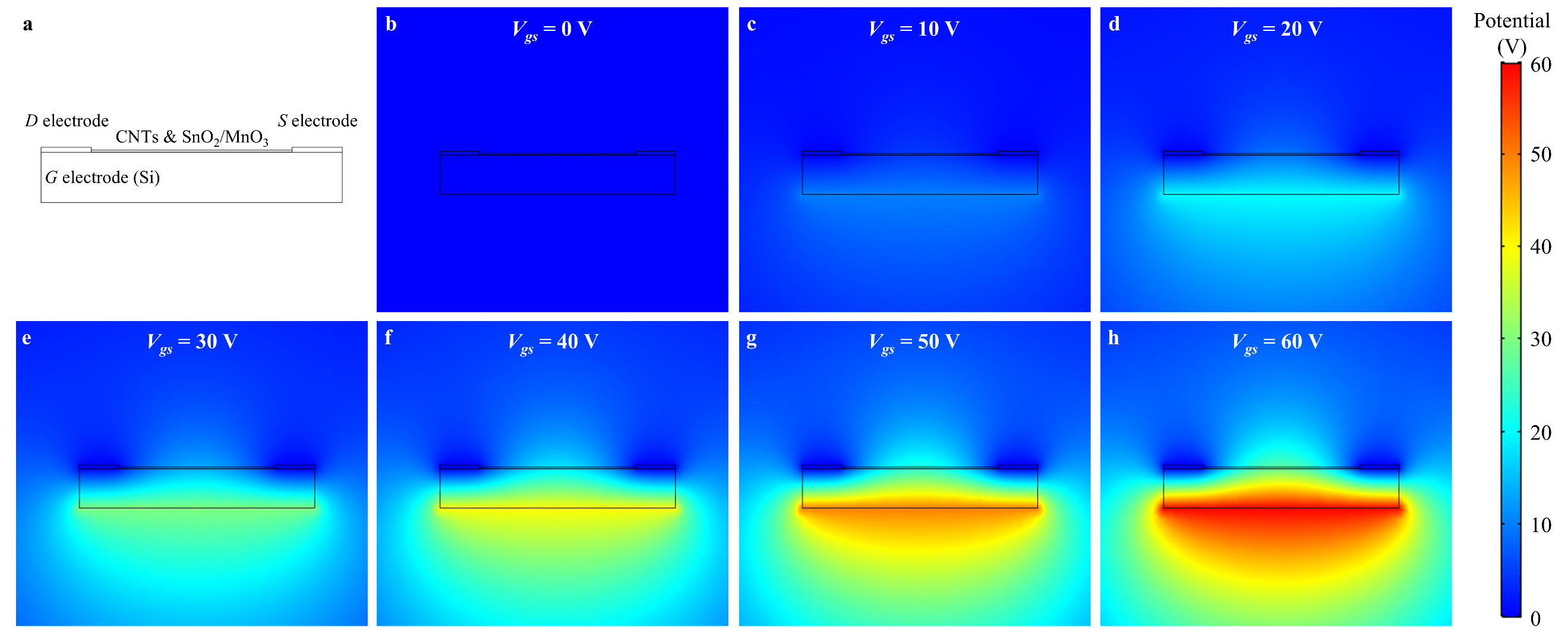


**Figure S8. The simulation diagram illustrates the potential distribution at different gate voltages *V*_gs_**. As the *V_gs_* increases, the potential applied to the sensitive material is intensified, even traversing through the substrate (p-doped Si), the dielectric layer (SiO_2_), and the channel (CNTs).


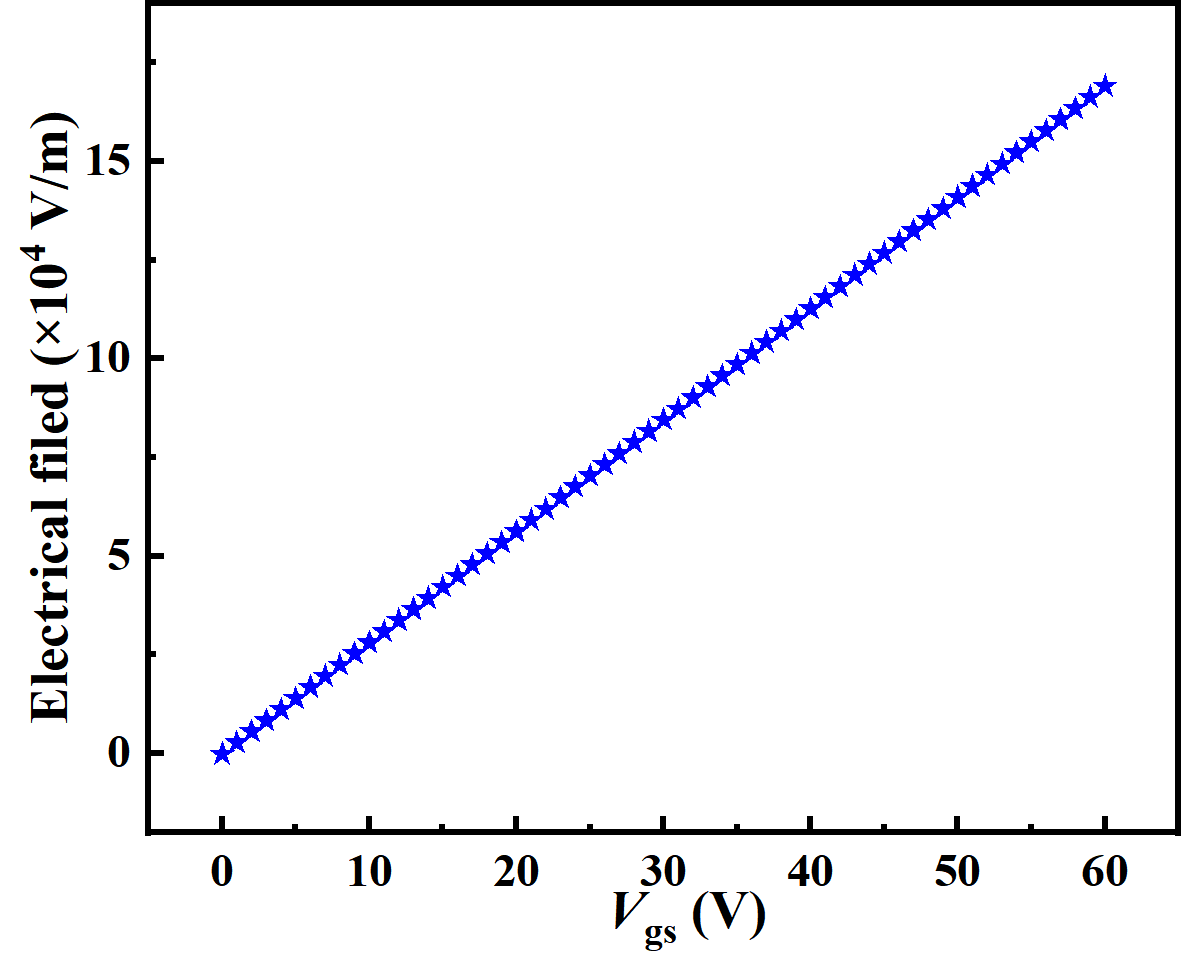


**Figure S9. The relationship between *V_gs_* and strength of electric field.**


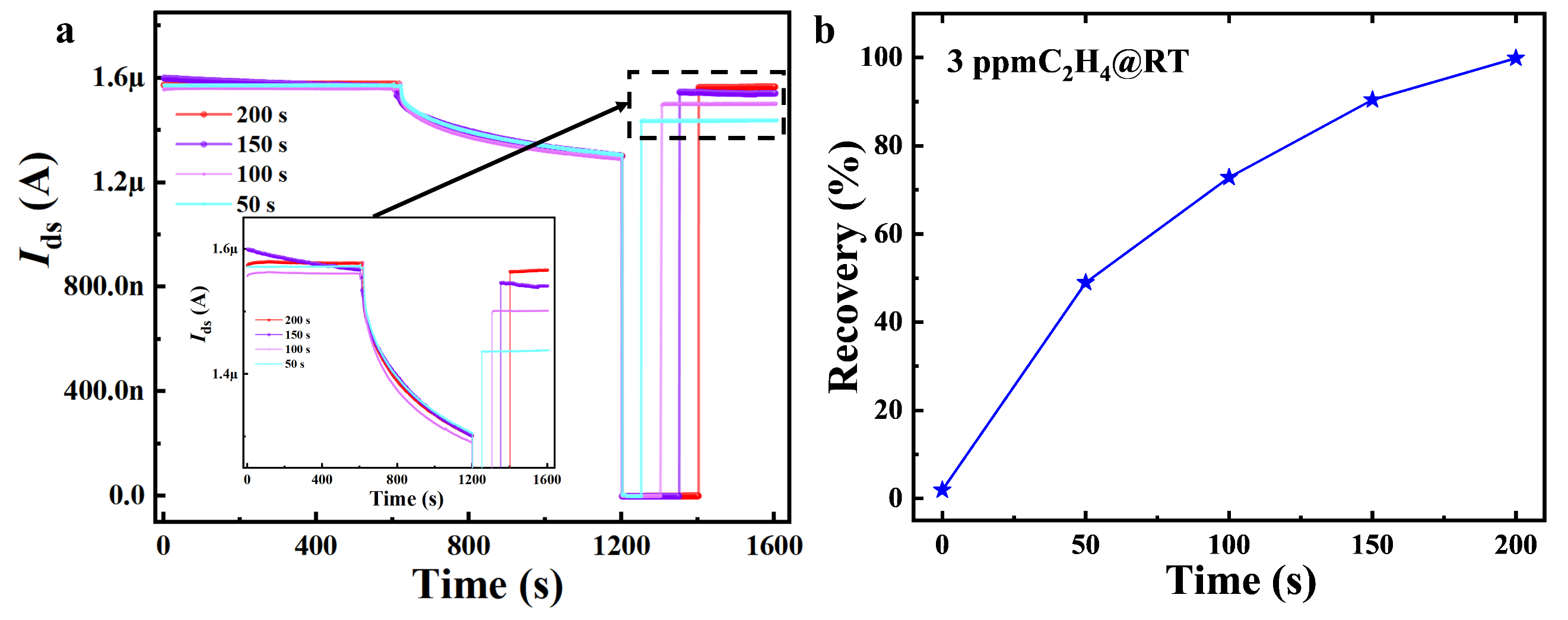


**Figure S10. The influence of the time applied +30 V pulse *V_gs_* on the recovery rate of the SnO_2_/MoO_3_ FET sensor.** (a) The dynamic response curve of the sensor to 3 ppm C_2_H_4_ exhibited under various gate voltages and (b) the relationship between the sensor's recovery time and the applied gate voltage. The recovery rate steadily increases over time, reaching 48.9% at 50 s and returning to approximately 99.8% of the baseline current at 200 s (in other words, the SnO_2_/MnO_3_ sensor can be completely recovered within 200 s).

**
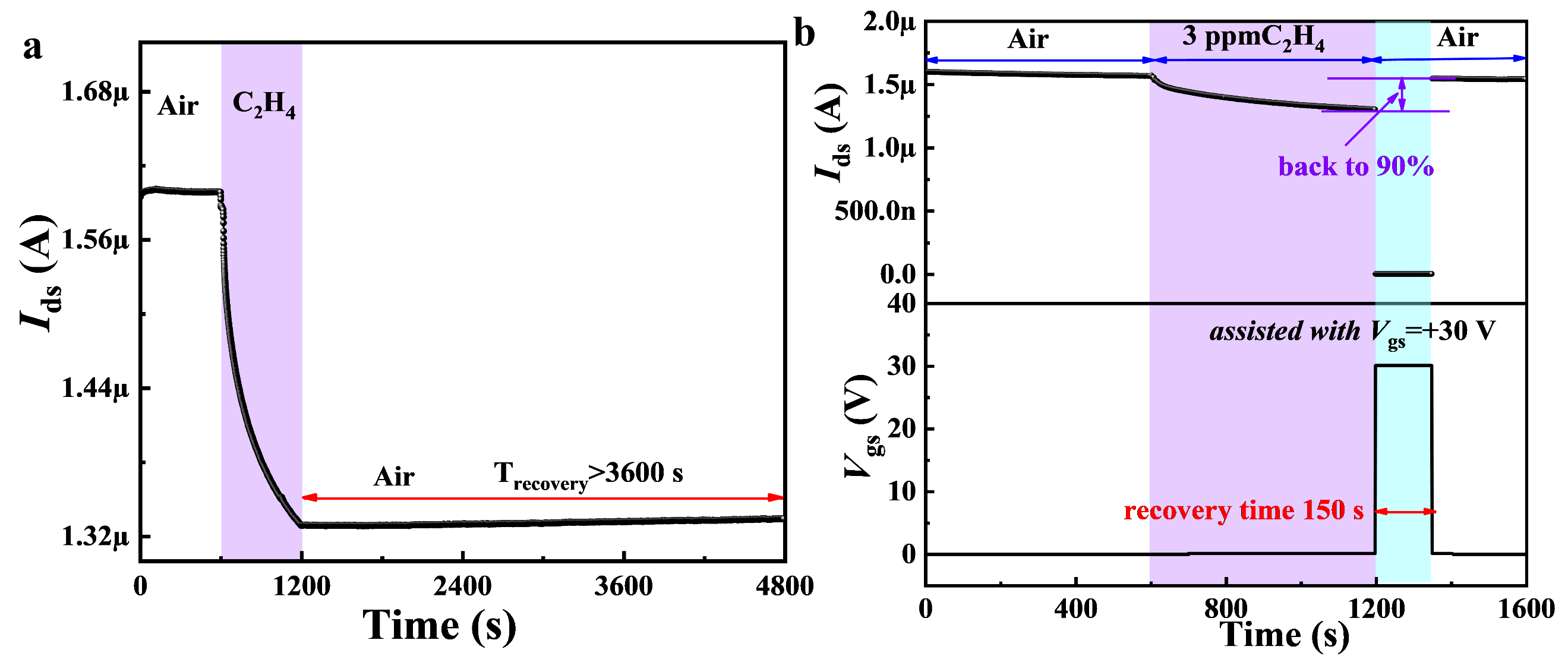
**

**Figure S11.** (a) The dynamic response of the SnO_2_/MoO_3_ FET sensor to 3ppm C_2_H_4_ without electrical field assisted (*viz.* *V_gs_* = 0 V in this work). The recovery time of the sensor exceeds 3600 s. (b) The dynamic response of the sensor under a +30 V pulse *V_gs_*. During the gas desorption stage, the pulse *V_gs_* of +30 V is applied, and the recovery time of the sensor is reduced to 150 s.


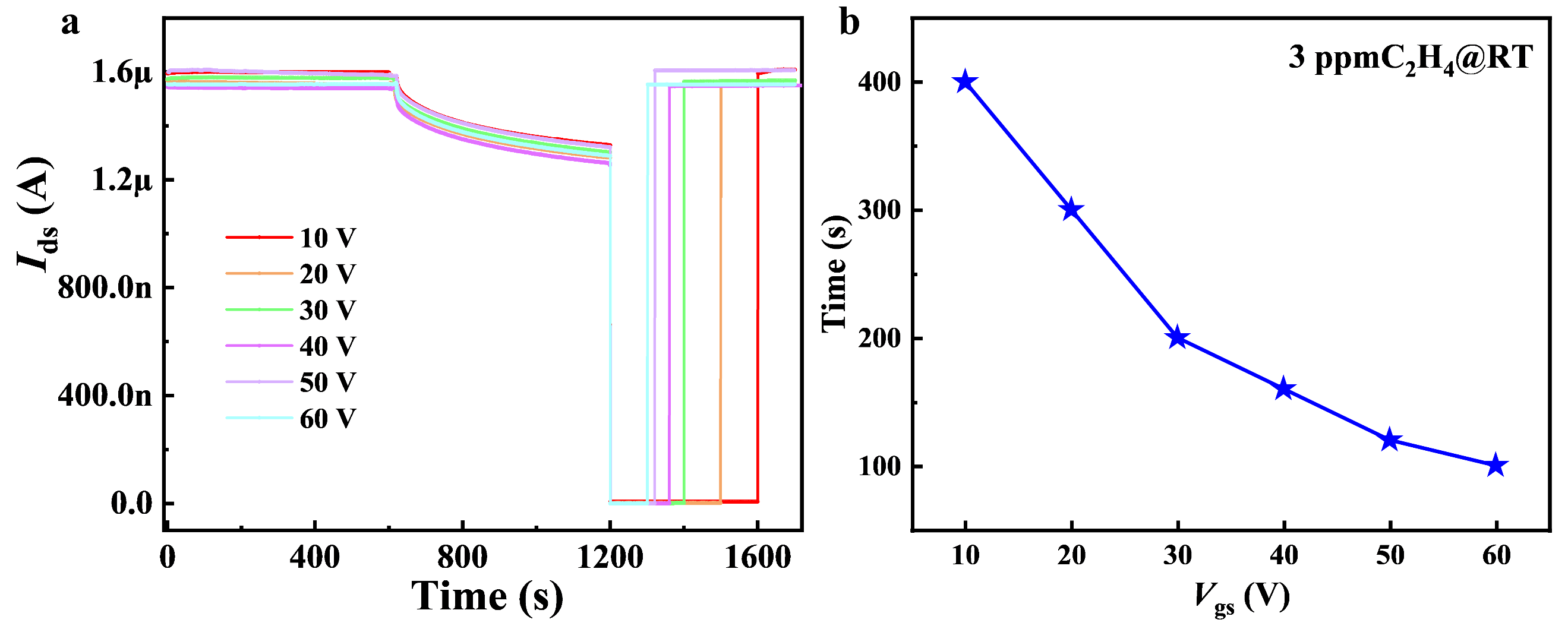


**Figure S12. The influence of the *V*_gs_ on the recovery time of the sensor.** (a) The dynamic response-recovery curve of the sensor to 3 ppm C_2_H_4_ under different *V_gs_*. (b) The relationship between the times required for the sensor to completely recover and the applied *V*_gs_. With a +60 V pulse *V_gs_* applied, the sensor completely recovers to its baseline in just 100 s.


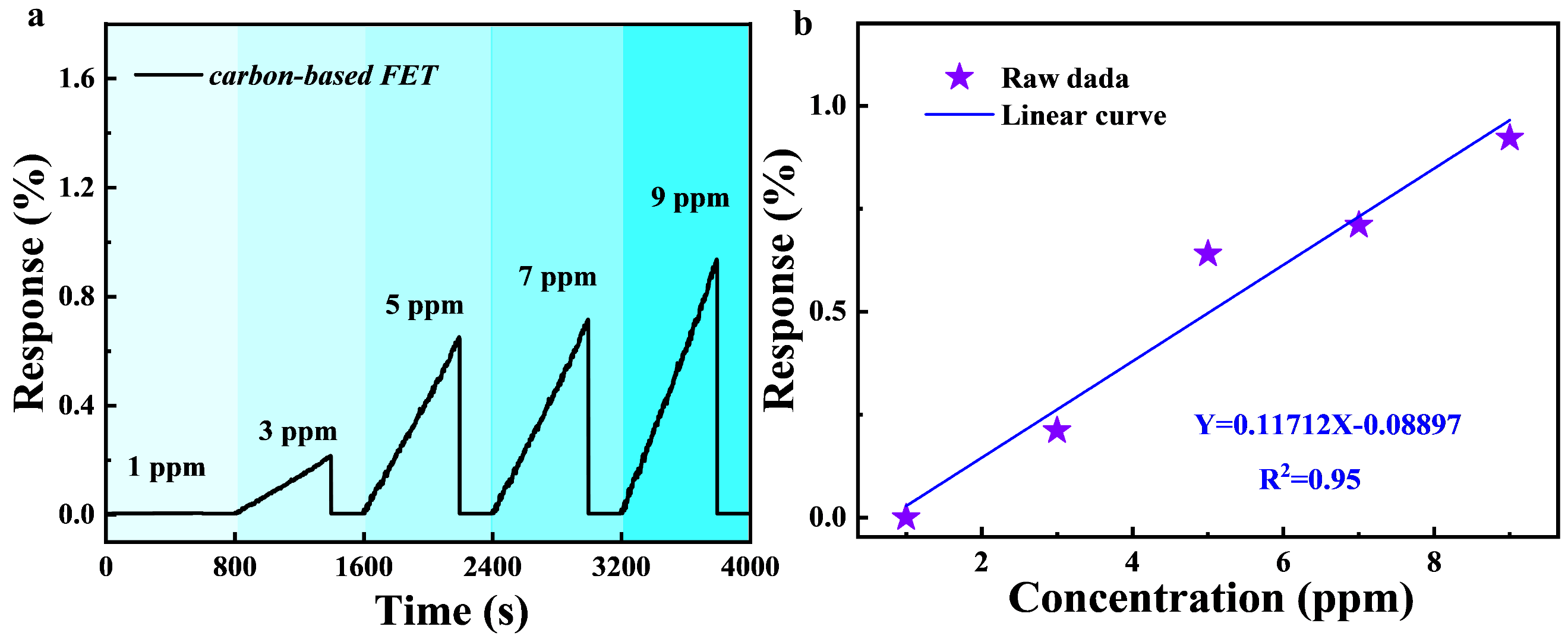


**Figure S13. C_2_H_4_ sensing performance of the carbon-based FET.** (**a)** The dynamic response and recovery curve of the carbon-based FET to 1-9 ppm C_2_H_4_. The injection durations for C_2_H_4_ and air are set to 600 s and 200 s, respectively, which aligns with the parameters established in the preceding experiment. The response to 9 ppm C_2_H_4_ is 0.92%. **(b)** The linear fitting curve for the response in the 1-9 ppm C_2_H_4_ concentration range, with a slope of 0.197%/ppm.


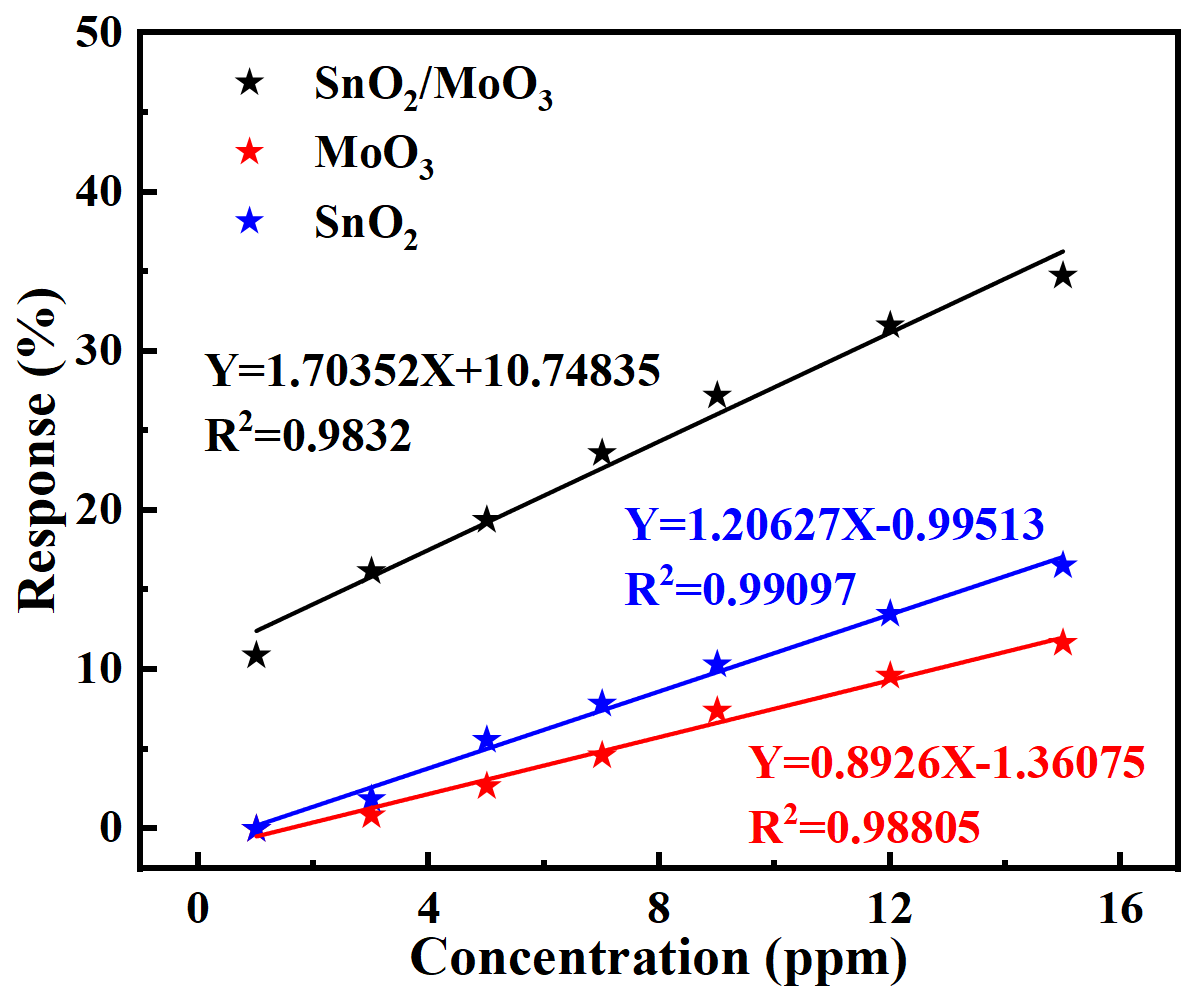


**Figure S14. The black, blue, and red curves in the figure represent the linear fitting for the response of the SnO_2_/MoO_3_ FET sensor, the SnO_2_ FET sensor, and the MoO_3_ FET sensor, respectively, over a concentration range of 1-15 ppm C_2_H_4_.** The linear fitting curve for the SnO_2_/MoO_3_ FET sensor can be expressed as Y = 1.70352X + 10.74835, with R^2^= 0.9832, where Y denotes the response value of the sensor, X denotes the C_2_H_4_ concentration, and R^2^ is the correlation coefficient. Compared to the other two sensors, it is evident that the SnO_2_/MoO_3_ FET sensor has a lower detection limit. In 1 ppm of C_2_H_4_, a response value of ~12% can be obtained, and the slope of the SnO_2_/MoO_3_ FET sensor is also larger than the other two sensors, indicating that the sensitivity of the SnO_2_/MoO_3_ FET sensor is higher, reaching 1.704 %/ppm.


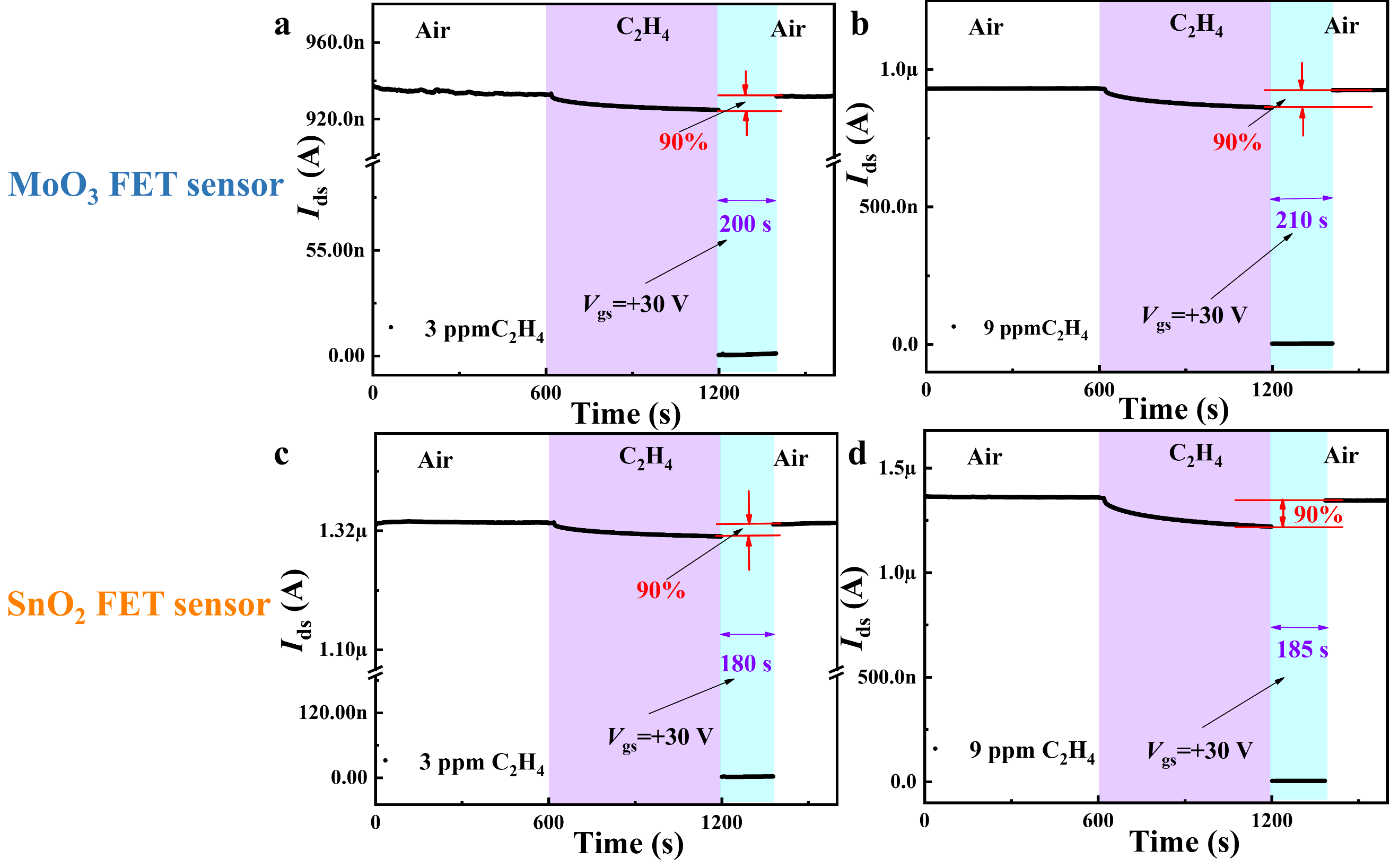


**Figure S15** **(a-b)** The dynamic response and recovery curve of the MoO_3_ FET sensor to 3 ppm and 9 ppm C_2_H_4_ under +30 V pulsed gate voltage. The MoO_3_ FET sensor exhibits a response of 0.86% and 7.42% to C_2_H_4_ concentrations of 3 ppm and 9 ppm, respectively. The MoO_3_ FET sensor exhibits a recovery time of 200 s when detecting 3 ppm C_2_H_4_ under the assistance of +30 V pulsed gate voltage. **(c-d)** The dynamic response and recovery curve of the SnO_2_ FET sensor to 3 ppm and 9 ppm C_2_H_4_ under +30 V pulsed gate voltage. The SnO_2_ FET sensor exhibits a response of 1.87% and 10.34% to C_2_H_4_ concentrations of 3 ppm and 9 ppm, respectively. The MoO_3_ FET sensor exhibits a recovery time of 180 s when detecting 3 ppm C_2_H_4_ under the assistance of +30 V pulsed gate voltage.


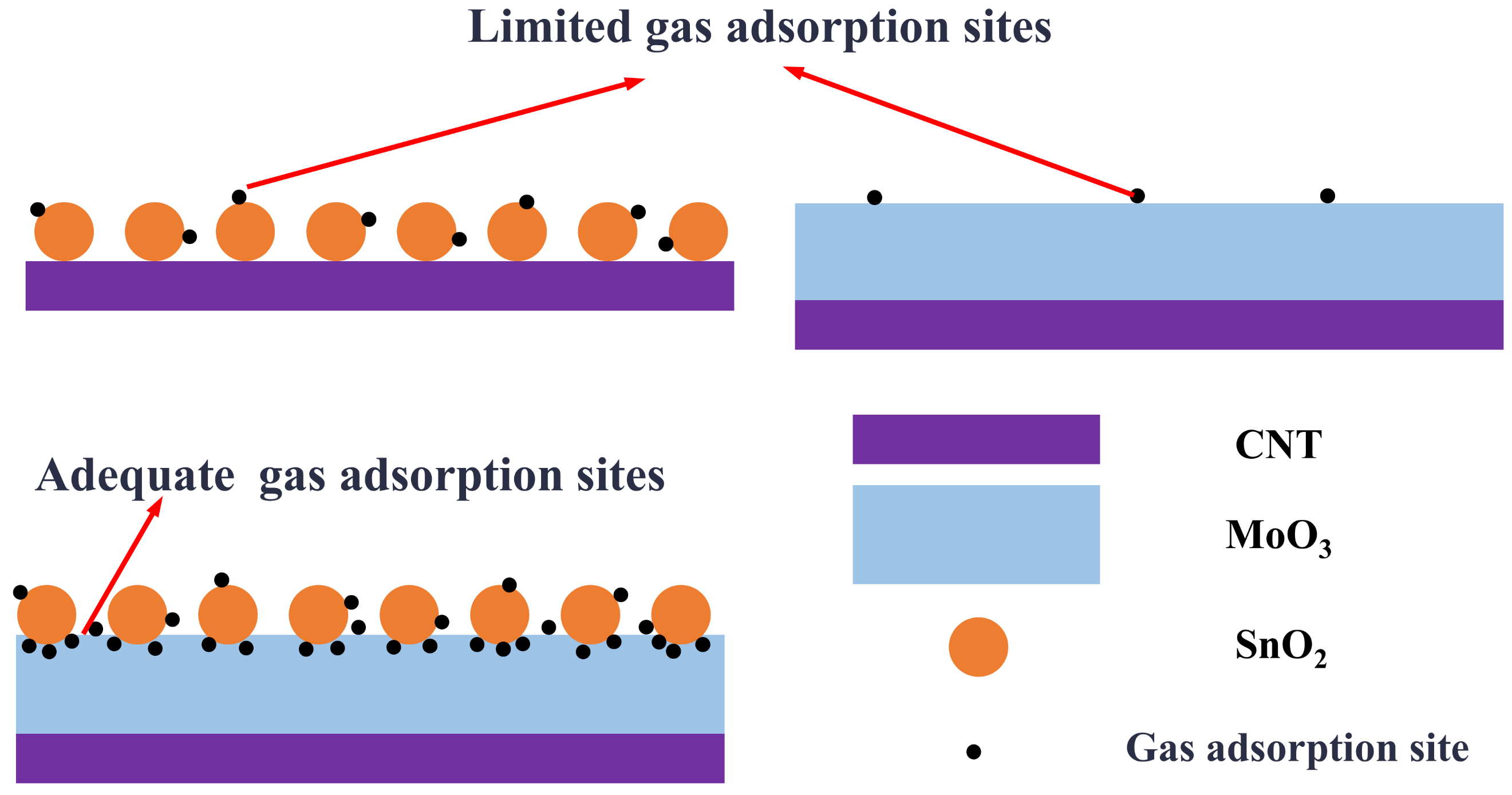


**Figure S16 Schematic diagram of the enhanced gas sensing performance of the SnO_2_/MoO_3_ FET sensor in comparison to MoO_3_ FET sensor and SnO_2_ FET sensor**. Upon combining SnO_2_ and MoO_3_, electrons transfer from SnO_2_ to MoO_3_. When the carrier concentration reaches equilibrium, the energy bands of the interfacial material bend, establishing an internal electric field within the interfacial region. ^[S2]^ This bending of SnO_2_ and MoO_3_ energy bands, coupled with the formation of the interfacial electric field, increases the number of gas adsorption sites and ultimately enhances sensitivity to C_2_H_4_.


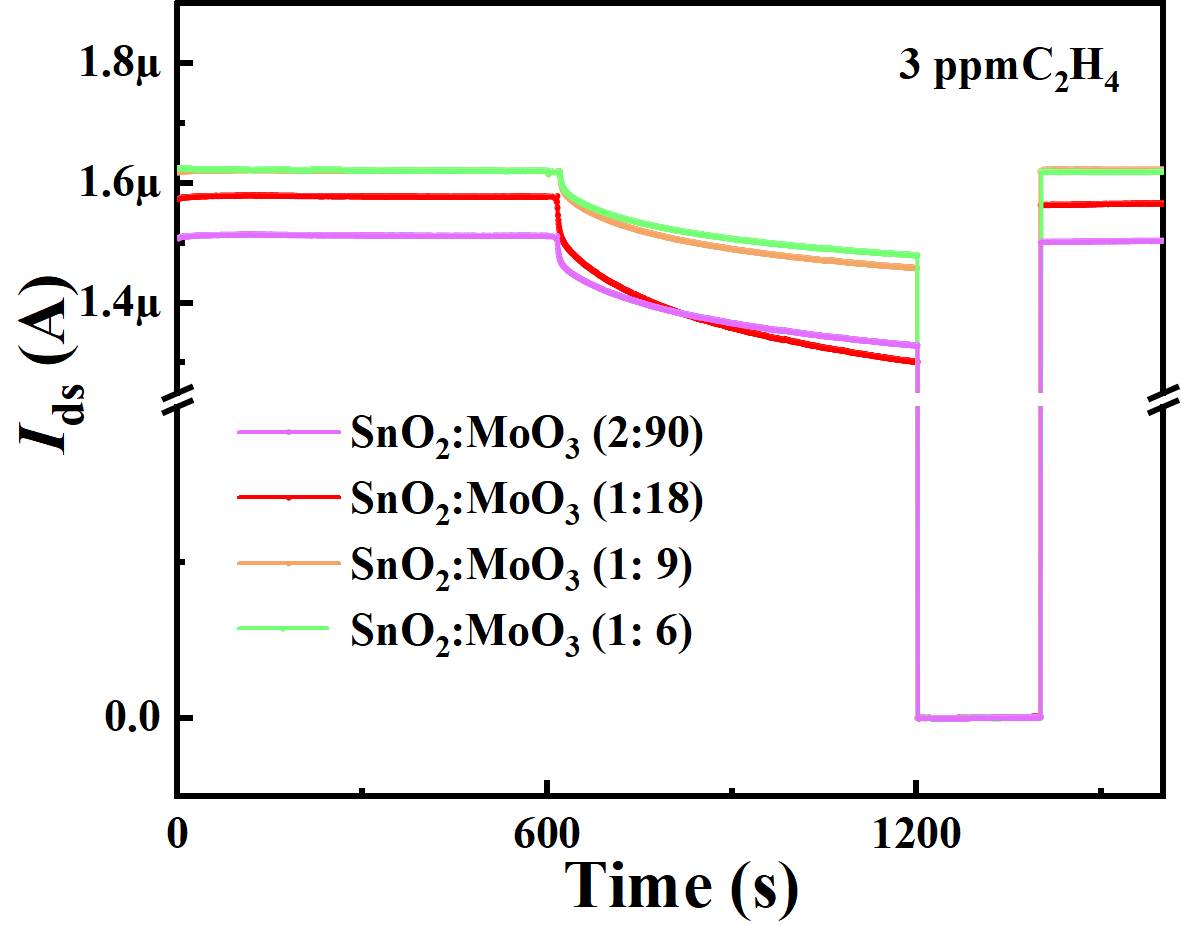


**Figure S17. The gas sensing performance of SnO_2_/MoO_3_ FET sensor with different SnO_2_:MoO_3_ molar ratio (2:90, 1:18, 1:9, and 1:6, corresponding mass fractions of SnO_2_ are:2.24 wt%, 5.45 wt%, 10.35 wt%, 14.77 wt%, respectively).** As the mass fraction of SnO_2_ increases from 2.24 wt% to 5.4 wt5%, the SnO_2_/MoO_3_ FET sensor’s response to 3 ppm C_2_H_4_ rises from 12.04% to 16.2%. This enhancement is attributed to the formation of a greater number of heterojunctions as the SnO_2_ concentration increases. However, as the mass ratio of SnO_2_ increases continuously, the sensor's response decreases. This decline is attributed to the excessive concentration of SnO_2_, which leads to aggregation and consequently reduces the contact area between C_2_H_4_ and the SnO_2_/MoO_3_ interface.


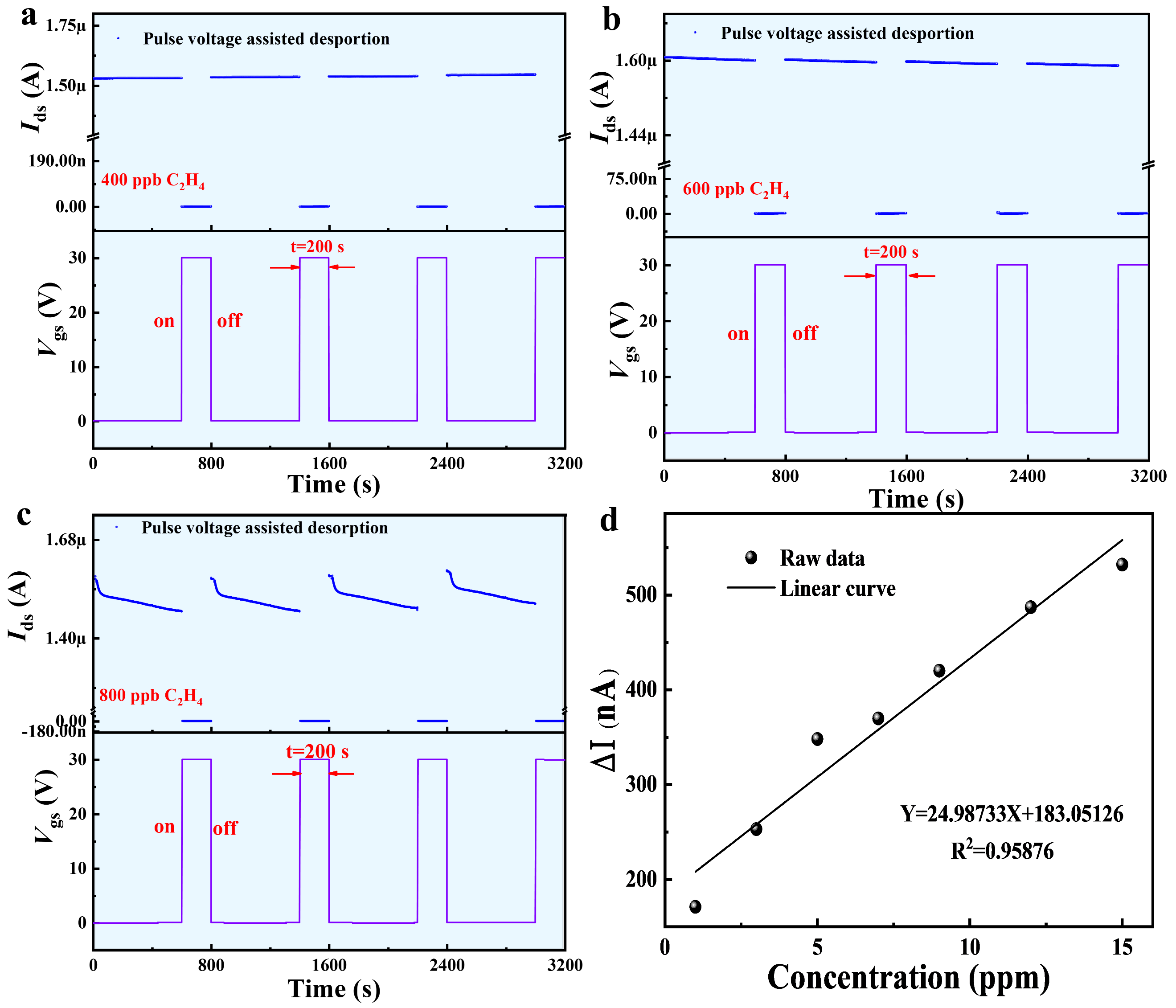


**Figure S18. The derivation of both the actual and theoretical lower detection limits for the SnO_2_/MoO_3_ FET sensor. (a)** 400 ppb. **(b)** 600 ppb. **(c)** 800 ppb. **(d)** Linear fitting curve of the current change value of the SnO_2_/MoO_3_ FET sensor to C_2_H_4_ concentrations ranging from 1 to 15 ppm.

At 400 ppb C_2_H_4_, the baseline current of the sensor remains stable with minimal fluctuation. When C_2_H_4_ concentration increases to 600 ppb, a slight decrease in the baseline current is observed, but the response < 1.5%. When the concentration reaches 800 ppb, a substantial change in the baseline current is observed, thereby establishing the lower detection limit of the sensor in experiments.

Except the experimental detection limit, the theoretical detection limit is calculated as:

$$\text{LOD =}\frac{\text{3×}\text{σ}}{\text{m}}\text{=}\frac{\text{3×0.835}}{\text{24.98733}}\text{=100.25 ppb}$$

The detailed derivation process is as follows: First, the noise deviation *σ* is determined to be 0.835 nA by analyzing the current fluctuation of the sensor in an air environment. Next, the slope of the linear fitting curve (m = 24.98733 nA/ppm) is obtained by fitting the current change curve of the sensor across various concentrations of C_2_H_4_ as illustrated in **Figure S18 d**. Finally, using a signal-to-noise ratio (SNR) of 3, which is a globally accepted standard, the theoretical detection limit of the sensor is derived.


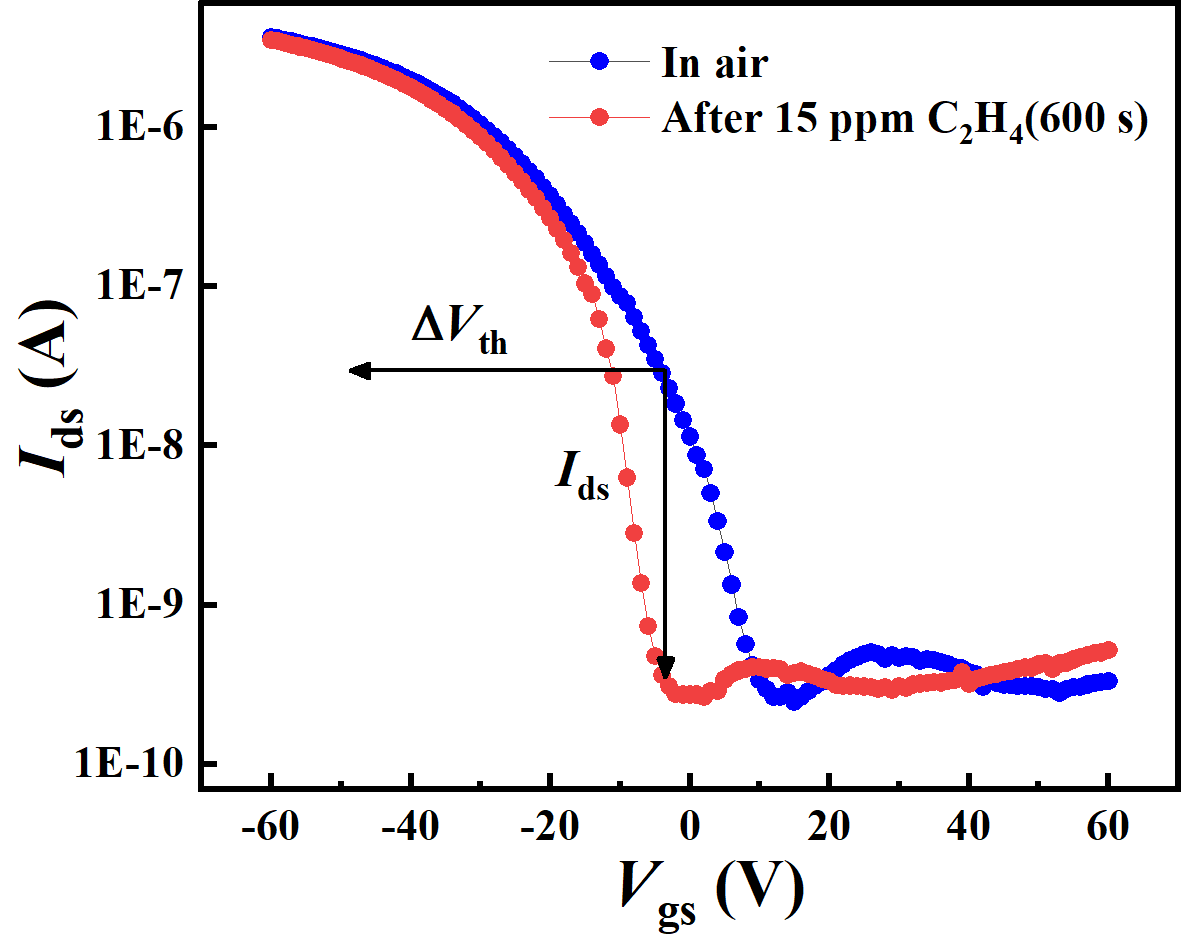


**Figure S19. Comparison of transfer characteristic curves of the SnO_2_/MoO_3_ FET sensor in air and versus in the 15 ppm C_2_H_4_.** After introducing 15 ppm C_2_H_4_ into the testing chamber, the transfer characteristic curve of the sensor shifts to the left due to changes in the FET work function resulting from electron transfer during C_2_H_4_ adsorption (the FET work function is positively correlated with the threshold voltage *V*_th_ of the FET).


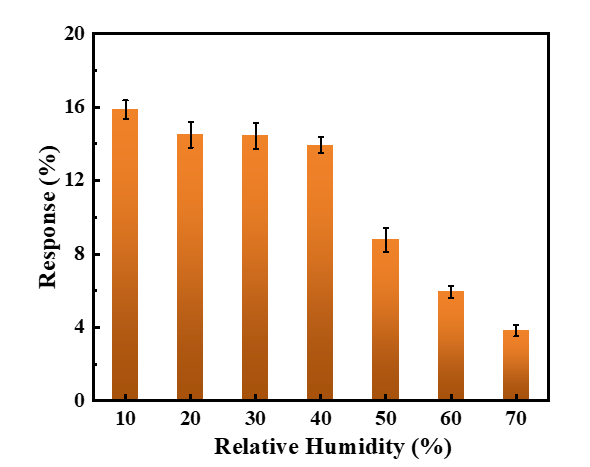


**Figure S20. The influence of humidity on the sensor response to 3 ppm C_2_H_4_, without electric field assistance.** When the relative humidity increases from 10% to 70%, the response decreases

from 15.87% to 5.83%.


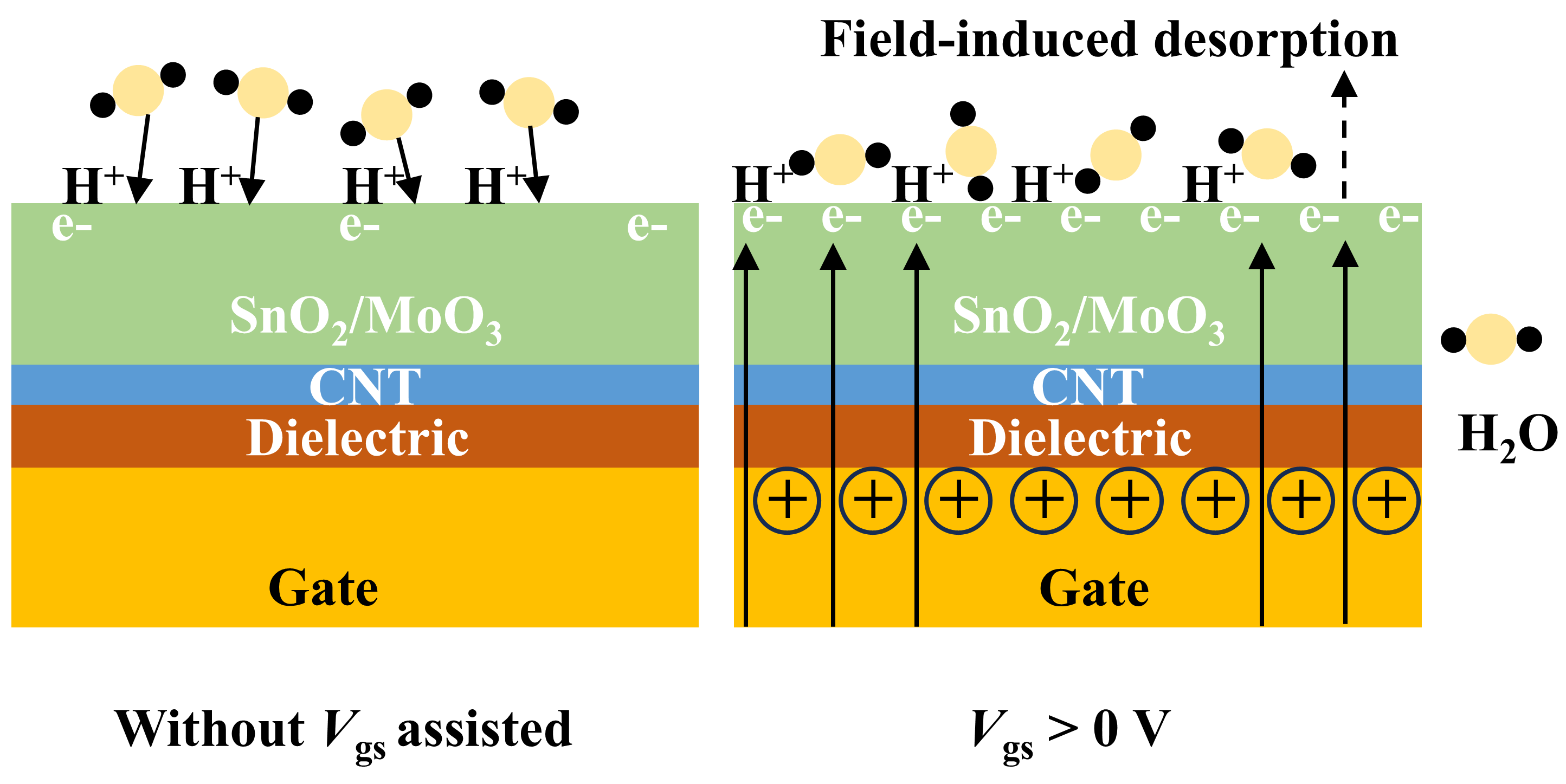


**Figure S21** **Schematic diagram of the mechanism of the electric-field-induced desorption of water molecules_._** The adsorption of water molecules on SnO_2_/MoO_3_ composite typically encompasses both physical adsorption and chemisorption. When chemisorption takes place, water molecules experience dissociative adsorption on the surface, leading to the formation of hydroxyl (-OH) groups and protons (H^+^). The dissociated H^+^ subsequently bind to surface oxygen anions, resulting in partially positively charged water molecules. When physical adsorption occurs, water molecules adhere to the material's surface through hydrogen bonding or van der Waals forces. Specifically, the hydrogen atoms with within the water molecules form hydrogen bonds with surface oxygen anions (O^2^⁻) or hydroxyl groups (-OH). This interaction enhances the partial positive charge characteristics of the water molecules. Upon application of a positive electric field by the sensor, free electrons accumulate on the surface of the SnO_2_/MoO_3_ material via electrostatic induction. The electron accumulation provides electrons to positively charged water molecules, thereby facilitating their desorption. ^[S3]^ Therefore, the SnO_2_/MoO_3_ FET sensor exhibits superior moisture resistance under pulsed gate voltage modulation.

**
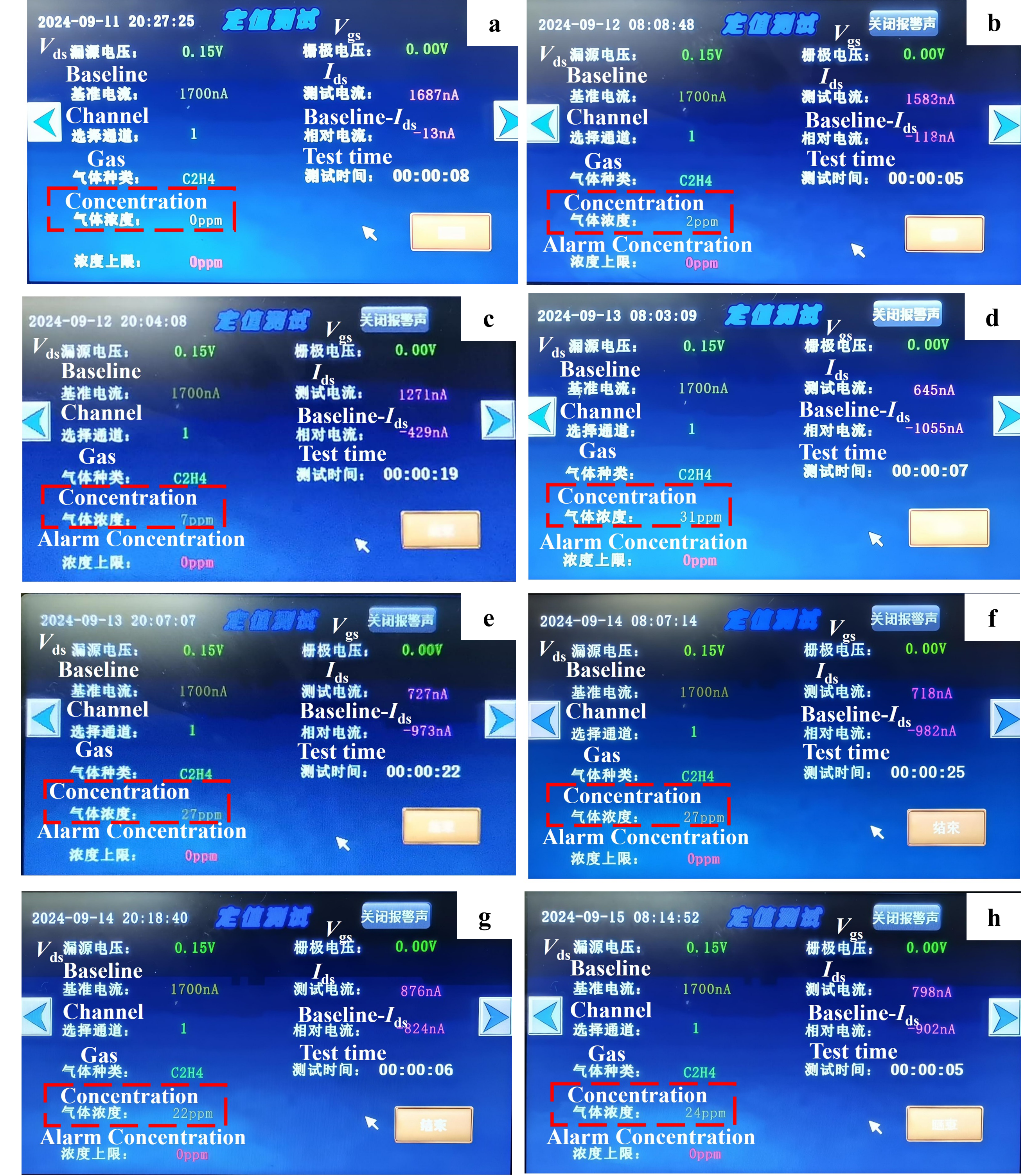
**

**Figure S22.** **The C_2_H_4_ concentration output from the** **portable test system during the banana’s ripe**. (a) 0 h. (b) 12 h. (c) 24 h. (d) 36 h. (e) 48 h. (f) 60 h. (g) 72 h. (h) 84 h. The tests are conducted every 12 hours, and the released C_2_H_4_ from bananas is recorded. The data shows that the C_2_H_4_ released from bananas increases from green to ripe, and decreases from overripe to rot.

**
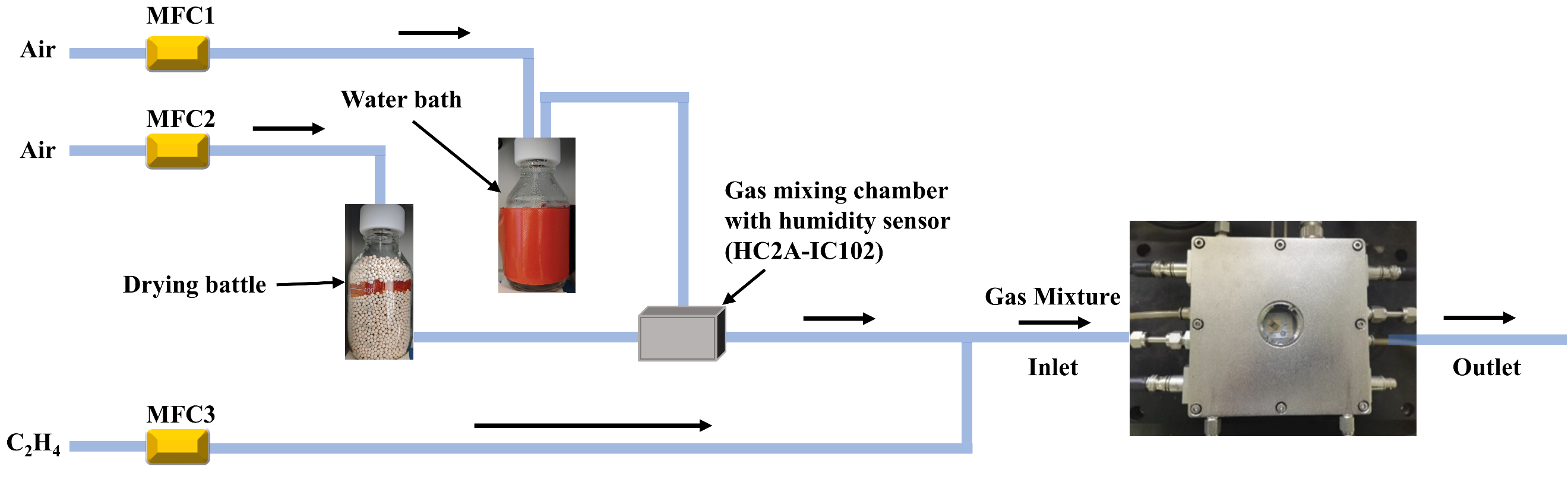
**

**Figure S23.** **Schematic diagram of the humidity environment introduced_._** In experiments, the humidity environment introduced is achieved via the dual-flow method. The MFC1 supplies humid air, the MFC2 supplies dry air. In accordance with the pre-established environmental humidity parameters, the system automatically adjusts the mixing ratio of MFC1 and MFC2 to achieve precise regulation of the target humidity. To ensure the accuracy of the mixing humidity, a high-precision humidity sensor (HC2A-IC102) is installed within the internal chamber. This sensor continuously monitors and provides feedback to controller to calibrate the humidity of the mixed air. Ultimately, the system will deliver the humidified air and C_2_H_4_ gas to the chamber at the predetermined ratio.


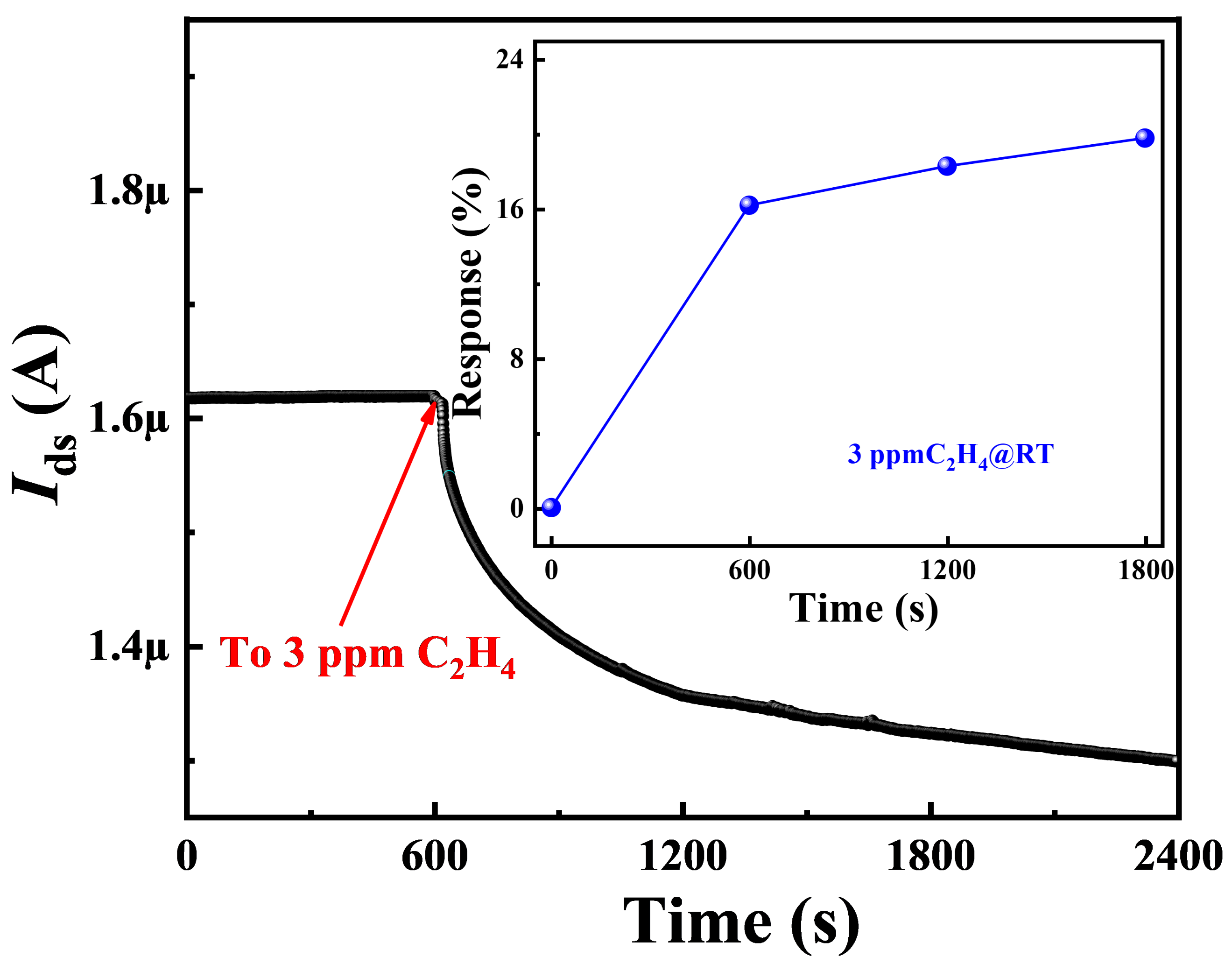


**Figure S24. Dynamic response curve of 3 ppm C_2_H_4_ sensor over 1800 s. The inset in the upper right corner illustrates the relationship between the sensor's response and the response time.** To validate the saturation of the SnO_2_/MoO_3_ FET sensor response curve, the dynamic response curve of the sensor is evaluated at a response time of 1800 s. Upon the introduction of 3 ppm C_2_H_4_ for 600 s, the sensor's response rapidly increased to 16.2%. As C_2_H_4_ is continuously added, the sensor gradually approached saturation state. Specifically, after introducing 3 ppm of C_2_H_4_ for 1200 s and 1800 s, the sensor responses are 17.3% and 18.04%, respectively. This indicates that the rate of change gradually decelerated, suggesting that the sensor response approached saturation around 600 s. During the gas sensing performance tests, a dynamic gas mixing method was employe. To enhance testing efficiency, we adopted a fixed time (600 s) for evaluating the gas sensing performance of the sensor.

**Supporting References**

[S1] J. Liu, Y. Xiang, Y. Chen, H. Zhang, B. Ye, L. Ren, W. Tan, A. Kappler, J. T. Hou, *Environ. Sci. Technol.* **57,** 12453-12464 (2023).

[S2] X. Liang, J. Zhang, L. Du, M. Z. Zhang, *Sens. Actuators B Chem.* **329** 129230 (2021).

[S3] J. K. Jeong, H. W. Yang, J. H. Jeong, Y.-G. Mo, H. D. Kim, *Appl. Phys. Lett.* **93,** 123508 (2008).
